# Breakage-fusion-bridge Cycles and Large Insertions Contribute to the Rapid Evolution of Accessory Chromosomes in a Fungal Pathogen

**DOI:** 10.1371/journal.pgen.1003567

**Published:** 2013-06-13

**Authors:** Daniel Croll, Marcello Zala, Bruce A. McDonald

**Affiliations:** Plant Pathology, Institute of Integrative Biology, ETH Zurich, Zurich, Switzerland; Duke University Medical Center, United States of America

## Abstract

Chromosomal rearrangements are a major driver of eukaryotic genome evolution, affecting speciation, pathogenicity and cancer progression. Changes in chromosome structure are often initiated by mis-repair of double-strand breaks in the DNA. Mis-repair is particularly likely when telomeres are lost or when dispersed repeats misalign during crossing-over. Fungi carry highly polymorphic chromosomal complements showing substantial variation in chromosome length and number. The mechanisms driving chromosome polymorphism in fungi are poorly understood. We aimed to identify mechanisms of chromosomal rearrangements in the fungal wheat pathogen *Zymoseptoria tritici*. We combined population genomic resequencing and chromosomal segment PCR assays with electrophoretic karyotyping and resequencing of parents and offspring from experimental crosses to show that this pathogen harbors a highly diverse complement of accessory chromosomes that exhibits strong global geographic differentiation in numbers and lengths of chromosomes. Homologous chromosomes carried highly differentiated gene contents due to numerous insertions and deletions. The largest accessory chromosome recently doubled in length through insertions totaling 380 kb. Based on comparative genomics, we identified the precise breakpoint locations of these insertions. Nondisjunction during meiosis led to chromosome losses in progeny of three different crosses. We showed that a new accessory chromosome emerged in two viable offspring through a fusion between sister chromatids. Such chromosome fusion is likely to initiate a breakage-fusion-bridge (BFB) cycle that can rapidly degenerate chromosomal structure. We suggest that the accessory chromosomes of *Z. tritici* originated mainly from ancient core chromosomes through a degeneration process that included BFB cycles, nondisjunction and mutational decay of duplicated sequences. The rapidly evolving accessory chromosome complement may serve as a cradle for adaptive evolution in this and other fungal pathogens.

## Author Summary

Chromosomal rearrangements are a hallmark of genetic differences between species. But changes in chromosome structure can also occur spontaneously within species, within populations, or even within individuals. The causes and consequences of chromosomal rearrangements affecting natural populations are poorly understood. We investigated a class of fungal chromosomes called accessory chromosomes that are not shared among all individuals within a species. Using a fungal pathogen possessing numerous accessory chromosomes as a model, we assessed chromosome diversity based on whole-genome sequencing and a PCR assay of chromosomal segments that included a global collection of isolates. We show that the accessory chromosomes are highly variable in their gene content and that geographic differences correlate with the number and the structure of the chromosomes. We applied the same approach to document chromosomal rearrangements occurring during sexual reproduction. We identified viable offspring carrying a novel chromosome that originated from a large duplication affecting the majority of the chromosome. Our study showed that chromosomal structure can evolve rapidly within a species to generate a highly diverse set of accessory chromosomes. This chromosomal diversity may contribute significantly to the adaptive potential of fungal pathogens.

## Introduction

Chromosomal rearrangements are major drivers of genome evolution. Dobzhansky [1] realized that chromosomal polymorphism would “supply the raw materials for evolution”, providing some of the earliest support for Darwin's theory of evolution. Since Dobzhansky's work on *Drosophila*, cytogenetic studies have revealed a large number of chromosomal rearrangements in the genomes of plant and animal species [2], including humans [3]. Chromosomal rearrangements were shown to contribute to sex chromosome differentiation [4], [5], reproductive isolation [6], speciation [7]–[10] and complex adaptive phenotypes [11].

Chromosomal rearrangements involve deletions, duplications, inversions and translocations within and among chromosomes. In most cases, the molecular mechanisms that generated the observed rearrangements are not known, but a common explanation is mis-repair of double-stranded DNA breaks [12], [13]. Repetitive DNA has been strongly associated with chromosome rearrangements in plant and animal genomes and is thought to promote non-allelic homologous recombination during meiosis due to the misalignment of dispersed repeats [14]–[16]. Telomeres play a major role in maintaining chromosome stability [17], [18]. Although chromosomes lacking a telomere are particularly susceptible to chromosomal fusion, subtelomeric double-strand breaks may also cause chromosomal fusion [19]. McClintock's classic cytogenetic work on maize in the 1930s and 1940s showed that mis-repair of damaged chromosomal ends could generate cycles of chromosomal degeneration termed breakage-fusion-bridge (BFB) cycles [20], [21]. BFB cycles begin when a telomere breaks off a chromosome. When the damaged chromosome replicates, its sister chromatids fuse and form a bridge during anaphase, with the two centromeres of the fused sister chromatids pulled into opposite poles of the dividing cell. After the bridge breaks, the resulting daughter cells receive defective chromosomes that lack telomeres and can initiate new BFB cycles. BFB cycles have also been identified in animals [22], [23] and yeast [24], [25]. In humans, BFB cycles play a significant role in cancer progression [18], [26], [27].

Fungal chromosomes are generally too small for traditional cytogenetic analyses based on chromosome staining and microscopic examination. But fungi were found to show extensive chromosomal polymorphisms following the invention of pulsed-field gel electrophoresis (PFGE). Application of PFGE revealed that many fungal species exhibit a high variability in chromosome number and size, even among individuals drawn from the same random mating population [28]–[30]. Mechanisms generating the differences in chromosome length and number remained largely elusive, although chromosome breakage and non-allelic homologous recombination among repetitive elements during meiosis were suggested to play a role [28], [31]. High chromosomal variability in pathogenic fungi may play an important adaptive role [32]. For example, dramatic changes in copy numbers of an arsenite efflux transporter in *Cryptococcus neoformans* occurred during experimental evolution favoring arsenite tolerance [33]. Chromosomal disomy was associated with increased antifungal drug resistance in several human pathogens including *C. neoformans* and *Candida albicans* [34], [35]. Copy-number variation and aneuploidy were frequently found in clinical and environmental isolates of the same species [36]–[39].

Some of the most polymorphic chromosomal complements were found in plant pathogenic fungi. Several species carry chromosomes that are not shared among all members of the species [40]. Chromosomes exhibiting a presence/absence polymorphism within a species have been referred to as B, dispensable, supernumerary or accessory chromosomes to differentiate them from the “core” chromosomes that are shared among all members of a species [29], [32], [40]. We refer to the chromosomes not shared among all individuals as accessory chromosomes because many of these chromosomes play an adaptive role in pathogen evolution, hence these chromosomes are not truly dispensable [32]. Nor do they fit the classic definition of B chromosomes, because they can carry many coding genes and may be necessary for survival in some environments. One of the best studied fungal accessory chromosomes was found in isolates of the pathogen *Nectria haematococca* and contains a gene cluster important for virulence on peas [41], [42]. The tomato pathogen *Fusarium oxysporum f. sp. lycopersici* contains several accessory chromosomes that carry a series of genes important for virulence [43]. In the rice blast fungus *Magnaporthe oryzae* and related species, a major effector called *AVR-Pita* that confers virulence on rice was frequently translocated between subtelomeric regions of different chromosomes including accessory chromosomes [44]. Flanking retrotransposons likely contributed to the extreme mobility of the *AVR-Pita* gene within and among closely related species.

The largest known complement of accessory chromosomes is found in the wheat pathogen *Zymoseptoria tritici* (syn. *Mycosphaerella graminicola* [45]). The eight smallest chromosomes of the reference genome of *Z. tritici*, ranging in size from 409–773 kb, were identified as accessory chromosomes [46]. The core chromosomes of the reference genome range in size from 1,186–6,089 kb [46]. In contrast to accessory chromosomes found in other pathogenic fungi, *Z. tritici* accessory chromosomes contain over six hundred annotated genes, however the function of these genes is poorly understood [46]. The fungus shows extensive chromosomal length and number polymorphisms within random mating field populations [30], [47]. Some of the chromosomal diversity appears to be generated through meiosis because progeny populations exhibited frequent chromosome loss and disomy as a result of nondisjunction of accessory chromosomes [48]. The origin of the accessory chromosomes of *Z. tritici* is not known, though both horizontal chromosome transfer from an unknown donor and degeneration of core chromosomes have been proposed [46]. Comparative genomics of closely related species suggested that several accessory chromosomes originated prior to the emergence of *Z. tritici* [49]. Several lines of evidence suggest that accessory chromosomes may be important for virulence, including the finding that genes on accessory chromosomes are under accelerated evolution and that these are more likely to show a protein signature consistent with a role in pathogenicity [46], [49].

The large set of accessory chromosomes in *Z. tritici* and its close relatives provides a powerful model system to elucidate the mechanisms underlying fungal chromosomal polymorphisms and the origins of accessory chromosomes. We combined population genomic resequencing and PCR-based chromosome segment genotyping to measure the diversity in chromosomal structure at a global scale. We then performed controlled sexual crosses to trace the fate of accessory chromosomes through meiosis and to identify structural rearrangements in chromosomes among the progeny. We confirmed the findings from our resequencing data with electrophoretic karyotyping that enabled chromosomal separation, isolation and visualization by Southern blotting. Our study provides the most comprehensive view to date of mechanisms underlying chromosomal polymorphisms in evolving fungal populations.

## Results

### Global populations are highly differentiated for presence or absence of chromosomal segments

**Figure 1:**
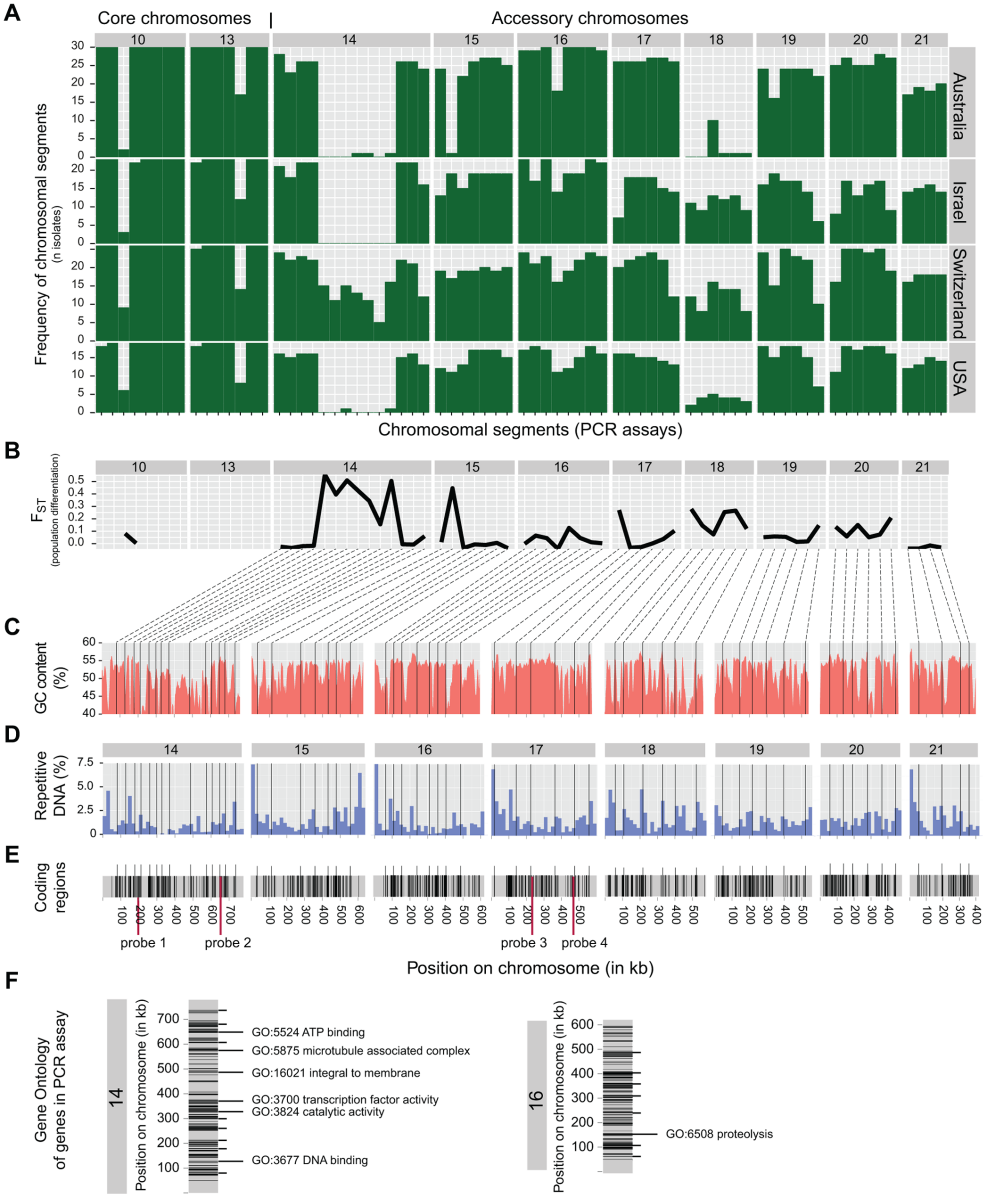
Global survey of diversity in accessory chromosomes of *Zymoseptoria tritici*. A) Presence or absence of chromosomal segments assayed by PCR in a global collection of four field populations located in Australia, Israel, United States and Switzerland (total n = 98). Green bars represent the number of chromosomal segments found within the populations. Core chromosomes 10 and 13 were included for comparison with the accessory chromosomes 14–21. B) Population differentiation based on the presence of chromosomal segments calculated by Wright's F_ST_. C) The physical location of each gene used for the PCR assays is shown on schematics drawn for each accessory chromosome. The variation of GC-content along the chromosomes is shown in red. D) Content of short direct repeats assessed in 20 kb segments. E) Location of coding regions according to the reference genome [46]. The location of probes used for Southern hybridizations to CHEF gels are indicated in red. F) Gene ontology terms for genes comprised in the PCR assay. Only genes on accessory chromosomes 14 and 16 were described by gene ontology terms [46].

*Z. tritici* is distributed globally and exhibits high genetic diversity for neutral markers [50]–[52] as well as high phenotypic diversity for quantitative traits, including virulence and thermal adaptation [51]–[53]. To assess the composition and frequency of accessory chromosomes across global populations, we designed 57 PCR assays covering all 8 known accessory chromosomes found in the reference strain IPO323 (chromosomes 14–21; [46]). Amplicons ranging in size from 400–600 bp were targeted to coding regions and primer sites were chosen in conserved regions of each gene (Table S1). The genes comprised in the PCR assay were evenly distributed along the accessory chromosomes and were located mostly in GC-rich regions interspersed by regions of higher repeat content (Figure 1C and 1D). Gene density varies along accessory chromosomes and the PCR assays covered the entire range of known gene locations for each chromosome (Figure 1E). The function of most genes included in the PCR assay is unknown and only 7 out of 57 genes were characterized by gene ontology (Figure 1F; [46]). As a control we designed 15 additional PCR assays covering core chromosomes 10 and 13. Known microsatellite loci were included in each PCR as a positive control. In total, we surveyed 98 isolates sampled from a global collection of four wheat fields at 72 evenly spaced chromosome positions (Table S2): Oregon, United States (n = 19), Israel (n = 23), Australia (n = 30) and Switzerland (n = 26).

The PCR assays on the core chromosomes 10 and 13 showed that 10 chromosomal segments were present in all 98 isolates (Figure 1A). Three chromosomal segments were missing in 1–3 isolates distributed at random across the populations. One segment on each chromosome was missing in a large fraction of the isolates, but was at approximately the same frequency across all populations (Figure 1A). None of the isolates was missing an entire core chromosome. In contrast, chromosomal segments on accessory chromosomes showed large frequency variations among populations and different accessory chromosomes showed different patterns of segmental presence/absence (Figure 1A). With the exception of chromosome 18, all accessory chromosomes were found at a frequency higher than 50% in the four field populations. Chromosome 16 was present at the highest frequency with several chromosomal segments being fixed within populations.

Individual accessory chromosomes showed substantial differences compared to the chromosomes of the Dutch reference strain IPO323. Central chromosomal segments located on chromosome 14 were almost entirely missing in isolates from Australia, the United States and Israel. Swiss isolates showed a central chromosomal segment at approximately half the frequency as segments closer to the telomeric ends of the chromosome. The haplotypic diversity for the presence or absence of individual chromosomal segments was substantial among isolates (Figure S1). Nearly every isolate showed a unique combination of presence or absence of individual accessory chromosome segments. We assessed the population differentiation for presence or absence of chromosomal segments among populations using Wright's F_ST_ statistic. Frequencies of several accessory chromosome segments were strongly differentiated among populations. The central segments of chromosome 14 showed F_ST_ ranging from 0.15–0.55 (Figure 1B). High levels of differentiation were also found for the second segment of chromosome 15 and the first segment of chromosome 17. Chromosome 18 showed elevated levels of differentiation across the chromosome, largely because this chromosome was almost entirely missing from the Australian and USA populations (Figure 1A). In contrast, previous data on neutral genetic markers on core chromosomes showed little differentiation among these and other populations [50].

### Population resequencing revealed variation in gene content among homologous chromosomes

**Figure 2:**
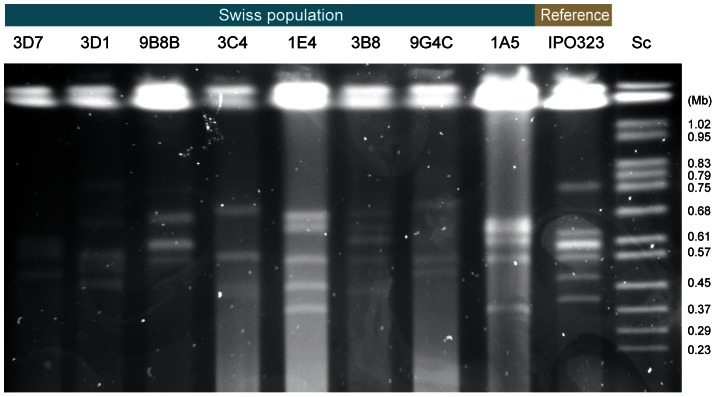
Electrophoretic karyotype diversity in accessory chromosomes among eight resequenced Swiss *Zymoseptoria tritici* isolates compared to the reference isolate IPO323. Chromosomal bands were separated by CHEF gel electrophoresis. The size marker is *Saccharomyces cerevisiae* chromosomes (Sc).

We found substantial karyotypic diversity in accessory chromosomes among isolates from Switzerland (Figure 2). In order to obtain a fine-scale map of structural variation in accessory chromosomes among isolates, we performed Illumina resequencing on 9 of the Swiss isolates (mapping coverage 10–23×; Table 1). We identified genomic divergence between the reference isolate IPO323 and the resequenced isolates by mapping all sequence reads to the finished reference genome. To avoid spurious read mapping in repetitive regions of the chromosomes, we restricted our comparison to the coding regions of the accessory chromosomes. Furthermore, we considered exons of multi-exon genes separately to avoid biases introduced by gene length. In summary, we mapped reads to 1763 exons corresponding to 654 unique genes. The average exon length on accessory chromosomes is 314 bp compared to 517 bp on core chromosomes.

**Table 1:** Illumina resequencing and assembly statistics of *Zymoseptoria tritici* isolates from the Swiss population.

Isolate	Number of reads	Coverage of reference genome (%)	Mean coverage at mapped positions (X)	Scaffold N50 (bp)	Longest scaffold (bp)	BioSample ID^1^	SRA sample ID
ST99CH3B8	6,145,362	87	11.11	109,481	475,584	[SAMN01815823]	[SRS383144]
ST99CH3C4	5′,709,810	88	10.17	98,927	493,063	[SAMN01815822]	[SRS383145]
ST99CH3D1	5,655,717	88	9.66	109,158	731,290	[SAMN01815821]	[SRS383146]
ST99CH3D7	5,814,117	86	10.96	136,993	539,729	[SAMN01815820]	[SRS383147]
ST99CH3F5	5,781,183	89	10.56	96,887	558,175	[SAMN01815819]	[SRS383148]
ST99CH1A5	8,114,322	89	15.79	91,984	665,994	[SAMN01815818]	[SRS383142]
ST99CH1E4	9,224,238	88	18.14	83,812	499,110	[SAMN01815817]	[SRS383143]
ST99CH9G4C	12,384,026	89	22.68	93,236	486,086	[SAMN01815816]	[SRS382972]
ST99CH9B8B	12,195,122	90	22.37	97,941	945,872	[SAMN01815815]	[SRS383009]

1 All samples were submitted under NCBI BioProject ID [PRJNA178194].

**Figure 3:**
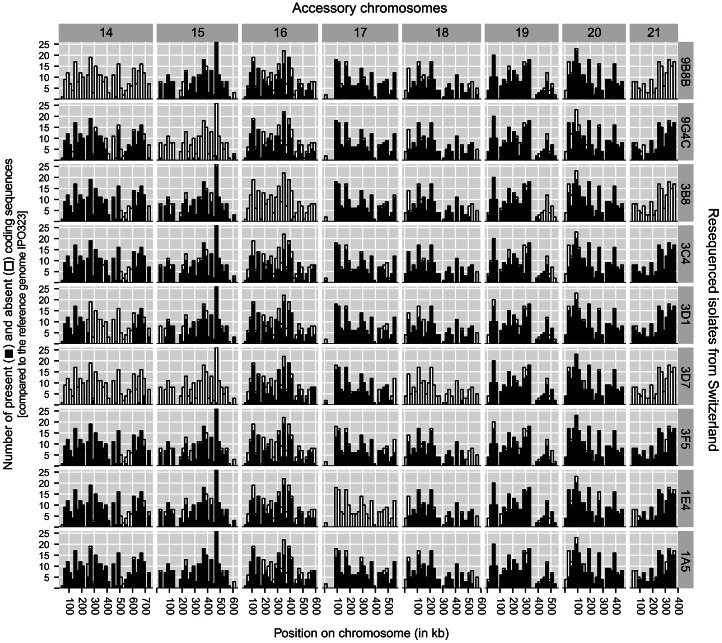
Genome resequencing of nine *Zymoseptoria tritici* isolates from a Swiss population. Illumina sequencing reads were mapped to annotated coding regions of the reference genome IPO323. The height of the bars represents the number of coding regions found in 20 kb segments in the reference genome. Horizontal rows show the nine different resequenced isolates. For each resequenced isolate black segments of the bars indicate the sum of present coding sequences per 20 kb segments. White segments of the bars indicate absent coding sequences.

The read depth from the resequencing data of 9 Swiss isolates mapped against the reference genome did not suggest any disomic chromosomes (i.e. doubled read depth for a particular chromosome). However, the different isolates varied greatly in gene content on accessory chromosomes. Four isolates (3C4, 3D1, 3F5 and 1A5; Figure 3) showed a nearly complete set of coding sequences compared to the reference genome, with a substantial number of coding sequences present on all 8 accessory chromosomes. Isolate 3D7 contained the smallest complement of accessory chromosome genes, as only four chromosomes showed a substantial proportion of coding sequences to be present. The read mapping to coding regions indicated that accessory chromosomes 14, 19 and 21 likely differ in length among homologous chromosomes (Figure 3). Chromosome 16 was found in all isolates except one. However, chromosome 16 likely differs substantially among isolates due to a large number of deletions compared to the chromosome 16 of the reference genome. Nearly all surveyed 20 kb segments along chromosome 16 showed missing genes in at least some of the resequenced isolates. The strongest variation in coding sequence complements was found among variants of chromosome 14. Isolate 3D1 lacked 149 out of 292 coding sequences, while smaller segments of missing coding sequences were found in six isolates (9G4C, 3B8, 3C4, 3F5, 1E4 and 1A5). The number of missing coding sequences ranged from 18–45 among these six isolates. At one end of chromosome 19, isolate 3B8 showed 46 missing coding sequences out of 220 coding sequences. Similarly, isolates 1A5 and 1E4 showed 31 missing coding sequences out of 155 coding sequences on chromosome 21.

### Chromosome 14 harbors a substantial length polymorphism gained through a recent insertion

**Figure 4:**
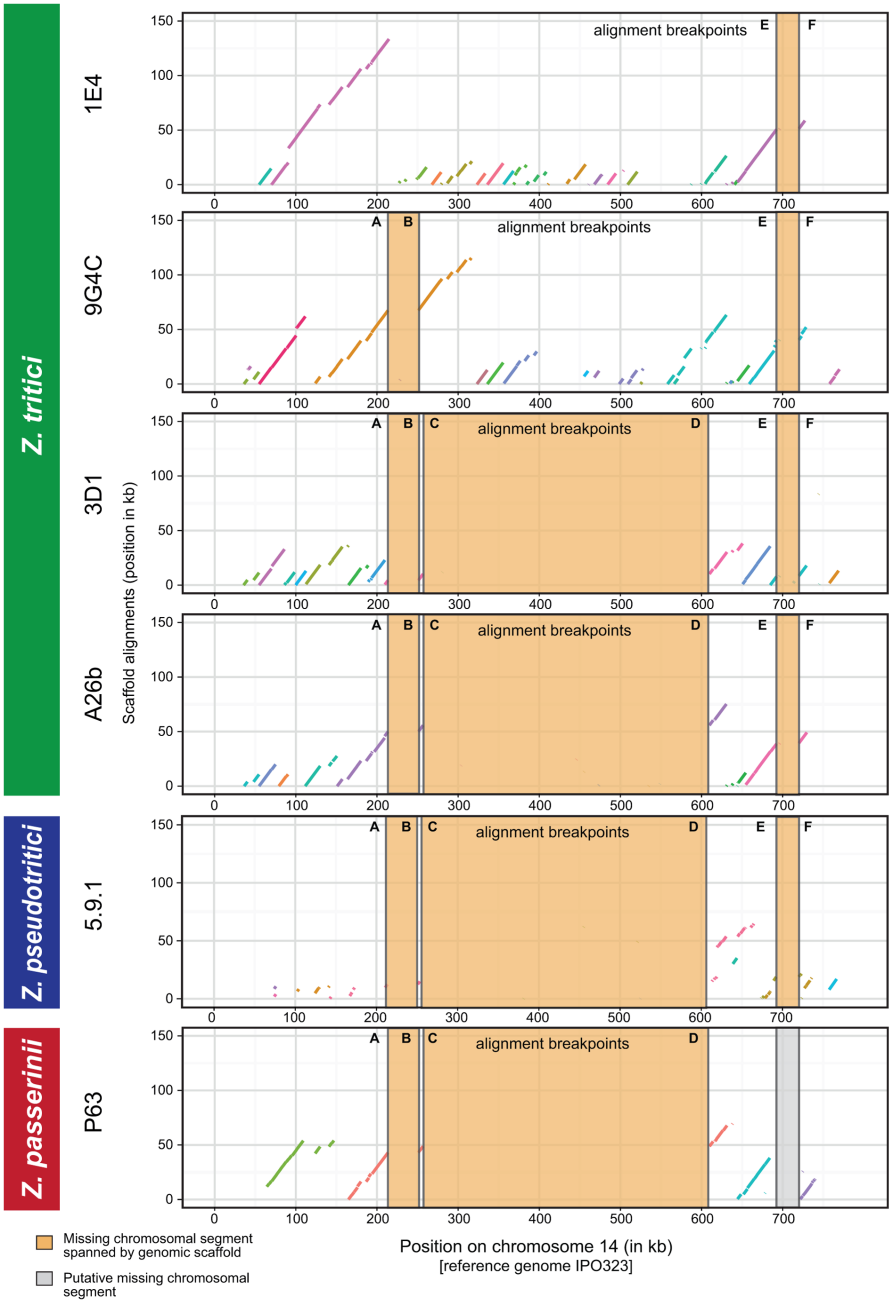
Comparisons of synteny among different variants of accessory chromosome 14 in related species. Scaffolds from *de novo* assemblies of the *Z. tritici* isolates 1E4 (n = 17), 9G4C (n = 16), 3D1 (n = 11) and A26b (n = 7) aligning to chromosomes 14 of the reference isolate IPO323 (horizontal axis) are shown. Scaffolds from isolates of related species *Z. pseudotritici* (5.9.1; n = 9) and *Z. passerinii* (P63; n = 4) aligning to chromosomes 14 of the reference isolate IPO323 are shown below. Orange bars show conserved deletions shared among different isolates and species in comparison to the reference chromosome 14. The grey bar indicates a putative deletion in *Z. passerinii* not spanned by a scaffold. Scaffolds are differentiated by color.

To investigate the nature of large missing chromosomal segments, we performed chromosome-length dotplots of the reference strain chromosome sequence against assemblies of the resequenced isolates. In particular we were interested in whether the large missing segments of chromosome 14 found in isolate 3D1 were due to a single deletion event. The comparison of the reference chromosome 14 of IPO323 with genomic scaffolds of resequenced isolates showed that both the Swiss isolate 3D1 and a previously sequenced Iranian isolate A26b carried one large deletion spanning nearly 400 kb (Figure 4). In addition, we identified two shorter deletions at homologous locations in both isolates (at 210–250 kb and 690–720 kb) compared to the reference chromosome 14. Interestingly, isolate 9G4C was lacking the large central deletion, however, this isolate shared the two peripheral deletions with isolates 3D1 and A26b (Figure 4). A fourth isolate (1E4) shared only the 690–720 kb deletion (Figure 4).

In order to determine the sequence of events leading to the large length polymorphism of chromosome 14 segregating within *Z. tritici* populations, we performed dotplots with genomic assemblies of three closely related species. We identified significant matching scaffold sequences from isolates of the closest relative *Z. pseudotritici* spanning the central deletions found in 3D1 and A26b (Figure 4). In the more ancestral species *Z. ardabiliae* we did not identify any significant matches for chromosome 14. However, in the more distantly related species *Z. passerinii*, a genomic scaffold spanned the entire central region. The deletion matched the regions identified in 3D1 and A26b, as well as *Z. pseudotritici* (Figure 4). This suggests that the ancestral chromosome 14 was significantly shorter than the chromosome 14 found in the reference strain IPO323. Furthermore, this finding indicates that the missing sequences in 3D1 and A26b actually represent large insertions into chromosome 14 of the reference strain.

**Figure 5:**
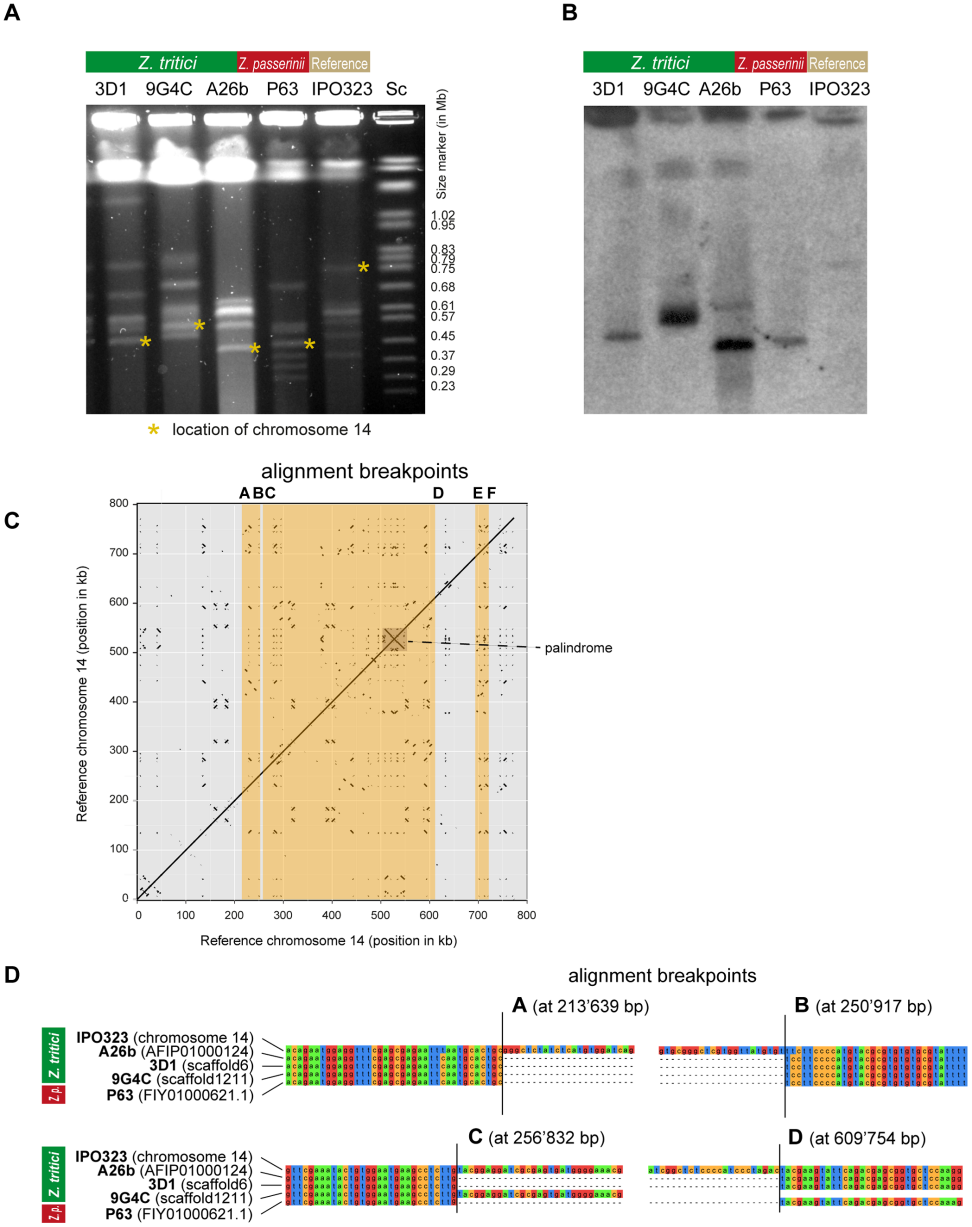
A) Pulsed-field gel electrophoresis of accessory chromosomes found in the *Zymoseptoria tritici* isolates IPO323 (reference isolate), 3D1, 9G4C, 26b and the *Z. passerinii* isolate P63. The size marker (Sc) represents chromosomes of *Saccharomyces cerevisiae*. Asterisks represent chromosome 14 variants identified by Southern hybridization with a chromosome specific probe. B) Southern hybridization with a chromosome 14 specific probe. C) Chromosomal dotplot of chromosome 14 in the reference genome of IPO323. Orange blocks indicate the identified deletions compared to other isolates of *Z. tritici* revealing a palindrome between 500 and 550 kb. D) Multiple sequence alignments of genomic scaffolds spanning breakpoints A–D are shown for *Z. tritici* isolates IPO323, A26b, 3D1 and 9G4C. In addition, we aligned the genomic scaffold found in the isolate P63 of *Z. passerinii*. Exact locations of the breakpoints A–D are shown in parentheses and refer to chromosome 14 of the reference genome IPO323.

We aimed to ascertain whether the predicted length variants of chromosome 14 are reflected in the karyotypic profiles of the different isolates. For this, we used chromosome-specific probes to identify chromosome 14 in different *Z. tritici* isolates and *Z. passerinii*. Hybridization with two chromosome-specific probes (see Table 2) located at opposite ends of the chromosomes showed that the reference isolate IPO323 carried a chromosome 14 in the size range of 780 kb (Figures 5A and 5B; data shown for probe 2) as expected for the isolate [46]. Isolates 3D1 and A26b both carried a substantially shorter chromosome 14 in the range of 400–450 kb, as predicted from the genomic scaffold alignments. The outgroup species *Z. passerinii* also carried a chromosome 14 that is substantially shorter than in IPO323 (Figures 5A and 5B). Isolate 9G4C was predicted to be of intermediate size between the variants found in IPO323 and 3D1 and A26b. Hybridization with chromosome-specific probes indeed identified a chromosome 14 variant of about 530 kb (Figures 5A and 5B; data shown for probe 2).

**Table 2:** Primer sequences used for the Southern hybridization probes specific for chromosome 14 and 17, respectively.

Chromosome	Probe	GeneID^1^	Forward primer (5′-3′)	Reverse primer (5′-3′)
14	probe 1	97558	TTT GGA ACA TCT GAC CAC GA	GCG CTG TTC TTC CAA GTA ATC
14	probe 2	111740	CAA GCA CCC TCT CAC AAA CA	AAG CCC GTA GGT TGG TAT TG
17	probe 3	97809	CTC TTG ACT TCC TCC ATT GTC AT	GTG AAT TGT TCG GGG AAG AG
17	probe 4	97838	CCA ATC CCA AGA AAA CCG	GAC CTT TTG TGA GCT TCT CAA GTA

1 GeneID refers to the reference genome annotation [46].

To better understand mechanisms leading to the sequence insertions, we identified the precise locations of the breakpoints by performing multiple sequence alignments of the reference chromosome 14 and the scaffold sequences of 3D1, A26b, 9G4C and *Z. passerinii*. Interestingly, the four sequence breakpoints characterizing the central section of chromosome 14 are at exactly homologous positions in *Z. tritici* and *Z. passerinii* (Figure 5D). The first set of breakpoints is located at 213,639 bp and 250,917 bp (breakpoints A and B on Figure 5D) in the IPO323 genome. The second set of breakpoints is located at 256,832 bp and 609,754 bp (breakpoints C and D on Figure 5D).

### Genomic characterization of the insertion in chromosome 14

**Figure 6:**
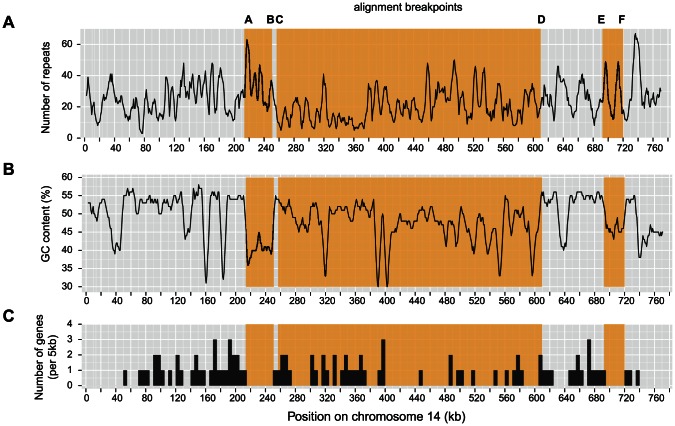
Genomic characterization of chromosome 14 in the *Zymoseptoria tritici* reference isolate IPO323. Orange shades indicate the alignment breakpoints identified among isolates of *Z. tritici*. A) Repeat density (for repeats up to a period size of 50 bp). B) GC content is shown in sliding windows with a window length of 5 kb. C) Numbers of genes are reported for each 5 kb window.

**Figure 7:**
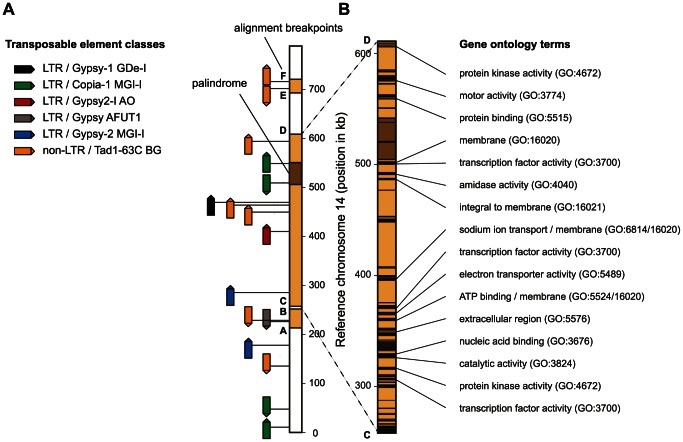
Remnants of transposable elements and predicted gene functions on chromosome 14 insertions. A) Identification of remnant long terminal repeat (LTR) and non-LTR transposable elements. Orange regions on chromosome 14 show the three inserted sequences flanked by alignment breakpoints A–F (as on Figure 4 and 5). The palindrome (shaded in brown; see Figure 5C) identified on chromosome 14 is flanked by two outwards facing Copia-1 type LTRs. Similarly, the inserted sequence at 700 kb (E–F) is flanked by two outwards facing non-LTR transposable elements. Related non-LTR transposable elements were also found near the flanking regions (A and D) of the large central insertion and the shorter insertion at 230 kb. B) Enlarged view of the large inserted sequence (between C and D) in chromosome 14. Black segments show the location of annotated genes. Genes with a functional prediction by Gene Ontology terms are indicated according to Goodwin et al. [46].

We aimed to identify the nature of the novel sequences inserted into chromosome 14 of the reference strain. The overall GC-content of chromosome 14 was 48.5% and corresponds to the lowest chromosomal GC content of the *Z. tritici* reference genome [46]. The two sequences located between breakpoints A-B and E-F showed a consistently lower GC-content than neighboring sequences (Figure 6). The largest sequence, located between breakpoints C and D, showed a heterogeneous GC-content. The density of repeat sequences increased sharply near the breakpoints of the shorter sequences located between breakpoints A-B and E-F (Figure 6). Furthermore, no genes were located between breakpoints A-B and only a single gene was found between breakpoints E-F (Figure 6). The large sequence inserted between breakpoints C-D contained several gene-poor regions. However, the overall gene density of this large sequence is similar to other regions of chromosome 14 (Figure 6). The large inserted sequence contained 16 genes with predicted functions related to a wide variety of metabolic, signaling and transcription factor activities (Figure 7B). By performing a self-alignment of the reference strain chromosome 14 sequence, we identified a substantial number of repeated sequences distributed along the chromosome. In particular, we found a large palindromic sequence located between 500–550 kb that showed high sequence similarity on both sequence strands (Figure 5C).

Chromosome 14 of the reference strain contains a series of transposable element (TE) remnants distributed along the chromosome (Figure 7A). Several of the inserted sequences contain TE remnants near the flanking regions. In particular, a non-long terminal repeat (non-LTR) element is found near both flanking regions of the insertion between alignment breakpoints E and F. The same element is found at flanking regions of the two other insertions (alignment breakpoints A and D). The large palindromic sequence is flanked by outwards facing LTR Copia element remnants.

### Distorted segregation of chromosomes in sexual crosses

**Figure 8:**
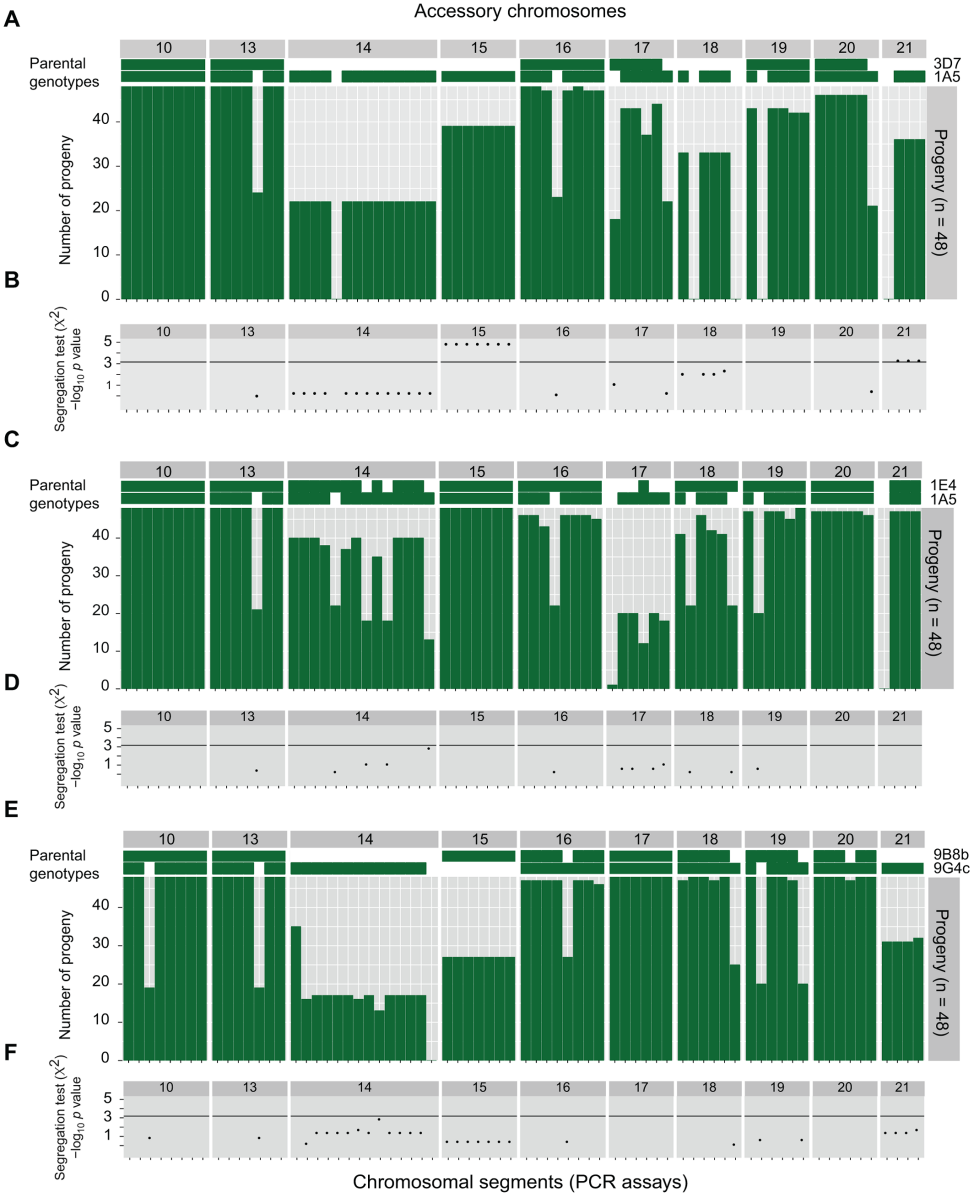
Variability in accessory chromosomes among progeny of three crosses between *Zymoseptoria tritici* isolates. A) Progeny of a cross between isolates 3D7 and 1A5 (Cross 3). Chromosomal segments of core chromosomes 10 and 13 and accessory chromosomes 14–21 were assayed by PCR. The two top rows indicate the two parental genotypes. The green bars show the number of individual chromosomal segments among the 48 progeny. B), D) and F) Test for random segregation of chromosomal segments that are present in only one of the two parental isolates. The −log_10_ transformed *p* values were corrected for non-independence and the horizontal bar represents the Bonferroni-corrected significance threshold (*p*<0.0007). C) Chromosomal segment numbers among 48 progeny from a cross between isolates 1E4 and 1A5 (Cross 2). E) Chromosomal segment numbers among 48 progeny from a cross between isolates 9B8B and 9G4C (Cross 1).

A major contribution to polymorphisms in accessory chromosomes may arise through meiotic recombination [48]. We performed controlled crosses involving three pairs of isolates from the Swiss population and analyzed 48 progeny from each cross. We applied the same PCR assays targeting 15 chromosomal segments on two core chromosomes and 57 chromosomal segments on the accessory chromosomes. Chromosomal segments on core chromosomes that were missing in either of the two parents were found to be segregating in approximately equal proportions in all three progeny sets (Figure 8). Patterns of segregation were different for several accessory chromosomes. In Cross 1 (9B8B×9G4C), we found a loss of chromosome 16 in one offspring despite the fact that both parental isolates were carrying a near full-length chromosome 16 (Figure 8E). In Cross 2 (1A5×1E4), we found that 8 progeny were missing all chromosome 14 segments, although both parental isolates carried the corresponding chromosome segments (Figure 8C). Similarly, chromosomes 16, 18, 20 and 21 were entirely missing from one offspring though both parents carried these chromosomes. Cross 3 (1A5×3D7) showed the strongest segregation distortions. Parental isolate 3D7 was missing four accessory chromosomes (Figure 8A; chromosomes 14, 15, 18 and 21). Two of these four chromosomes (15 and 21) were inherited in significantly higher proportion than expected under random segregation (*X* ^2^ test, p<0.0007 multiple comparisons corrected, Figure 8B). Interestingly, in Cross 1 the parental strains similarly differed in their presence of chromosomes 15 and 21, however we did not detect any significant segregation distortion in this cross (Figure 8E). Furthermore, two progeny of Cross 3 lost accessory chromosomes 17, 19 and 20 entirely, although both parental strains carried these chromosomes.

### Meiosis generates novel electrophoretic karyotype profiles

**Figure 9:**
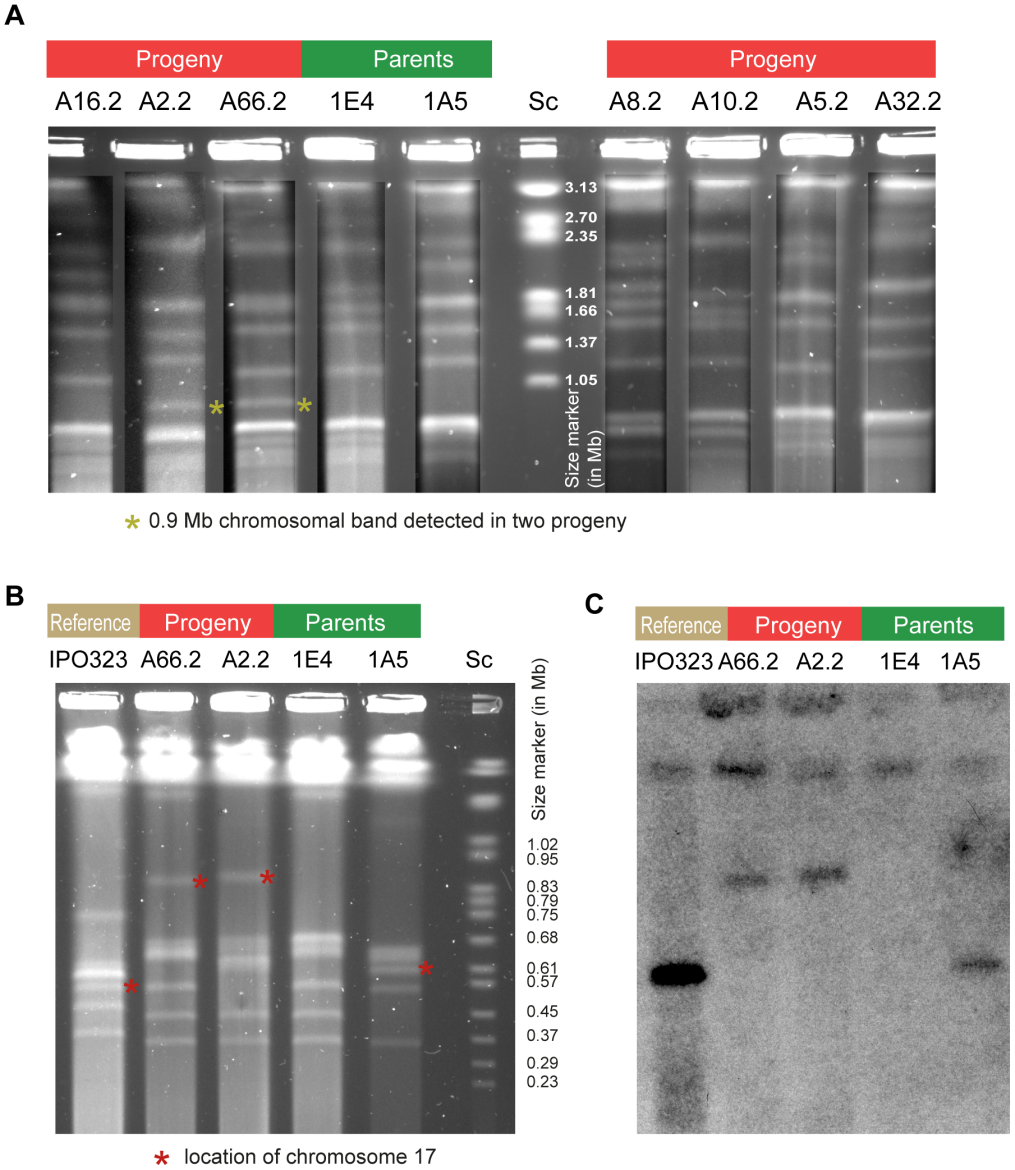
Pulsed-field gel electrophoresis of accessory chromosomes in the parental isolates 1E4 and 1A5, as well as their progeny A2.2 and A66.2. The isolate IPO323 was added as a reference. Both progeny showed a new chromosomal band at 0.9 Mb that is absent in either parental isolate and other screened progeny. Sc represents chromosomes of *Saccharomyces cerevisiae* added as a size marker. A) Pulsed-field gel electrophoresis of medium-sized chromosomes (up to approx. 3 Mb) of parental isolates 1E4 and 1A5 and 7 progeny. Progeny A2.2 and A66.2 showed a new chromosomal band at 0.9 Mb indicated by an asterisk. B) Pulsed-field gel electrophoresis of accessory chromosomes of parental isolates 1E4 and 1A5 and progeny A2.2 and A66.2. Asterisks identify chromosome 17 variants identified by hybridization. C) Southern hybridization of a chromosome 17 specific probe on chromosomes separated by pulsed-field gel electrophoresis of parental isolates, progeny and the reference isolate IPO323 as in B).

We randomly selected 24 and 34 offspring from Cross 1 and Cross 2, respectively, in order to identify changes in electrophoretic karyotype profiles among progeny. Progeny of both crosses showed substantial karyotypic diversity. Through hybridization with chromosome-specific probes, we found that parental isolates of Cross 2 showed length variation for chromosome 19 of approximately 0.3 Mb (data not shown). Chromosomes 15 and 21 showed nearly identical chromosome lengths among the parental isolates. Progeny of Cross 2 segregated the two length variants of chromosome 19 in approximately equal proportions (data not shown). Larger chromosomes (1.0–3.0 Mb) of parents and progeny of Cross 2 showed similarly diverse electrophoretic karyotypes (Figure 9A). In Cross 2, we identified two progeny (A2.2 and A66.2) out of 34 tested with a chromosomal band estimated to be around 0.9 Mb. However, neither of the two parents were found to have a chromosomal band in the range of 0.7–1.2 Mb, as shown by different PFGE gels optimized to separate either the smallest (<1.0 Mb) or medium-sized chromosomal bands (1.0–3.0 Mb) (Figures 9A and B).

### Genome resequencing and chromosomal hybridization reveals aberrant fusion event during meiosis

**Figure 10:**
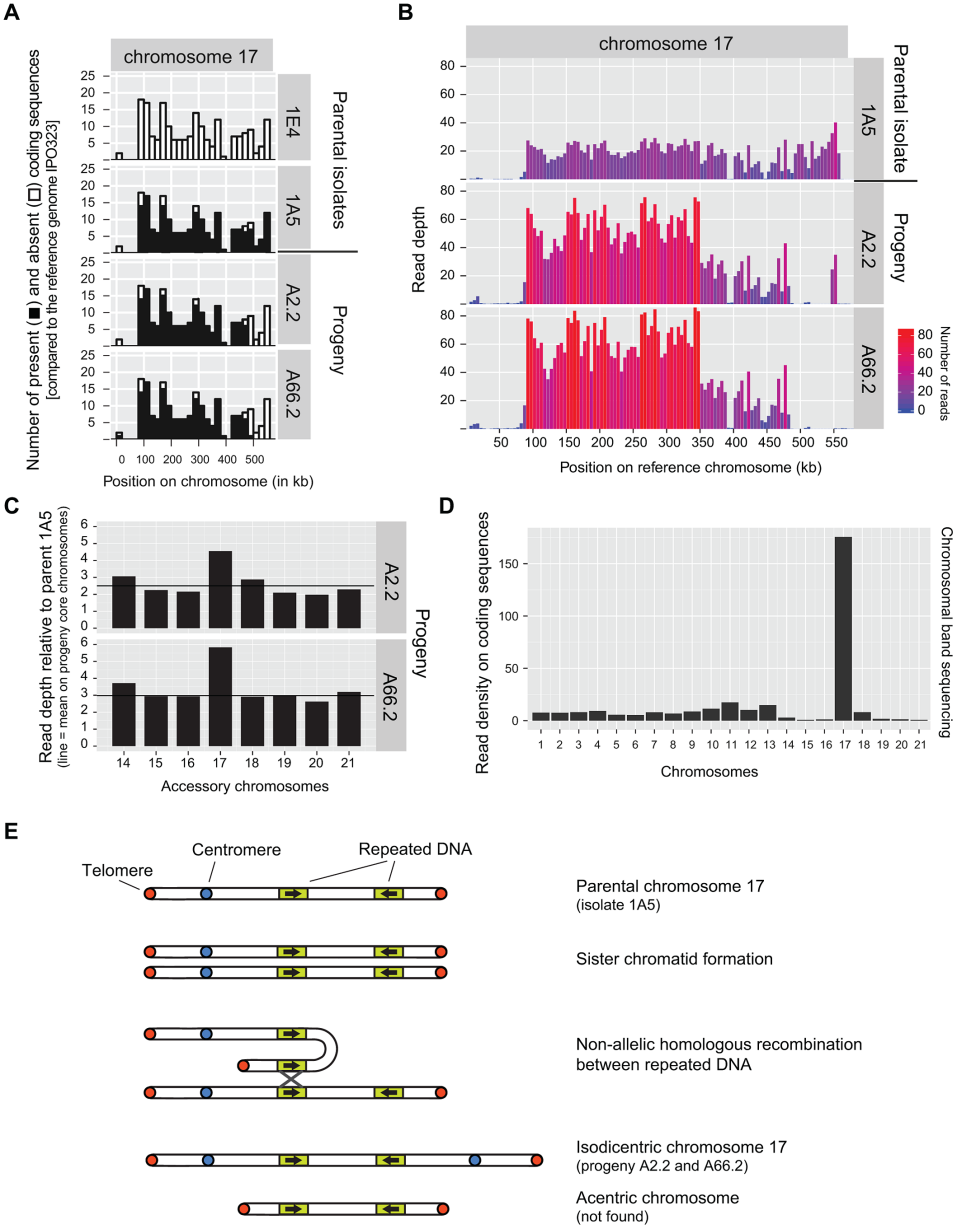
Initiation of a breakage-fusion-bridge cycle of *Zymoseptoria tritici* chromosome 17 during meiosis. A) Genome resequencing of two progeny (A2.2 and A66.2) from a cross between the parental isolates 1E4 and 1A5. Parent 1A5 carries accessory chromosome 17 (parent 1E4 is missing chromosome 17). Illumina sequencing reads were mapped to known coding regions of chromosome 17 on the reference genome IPO323. Black and white segments of the bars represent presence and absence, respectively, of particular coding sequences in 20 kb sections along chromosomes 17. B) Variation in read density along chromosome 17 of parental isolate 1A5 and progeny A2.2 and A66.2. C) Variation in read density among accessory chromosomes in the offspring A2.2 and A66.2. Illumina sequencing reads were mapped to coding sequences of the reference genome IPO323. Read density is reported as fold-difference between the offspring and the parental isolate 1A5. As a reference, the mean fold-difference on core chromosomes is reported as a horizontal line. Both offspring showed a near two-fold higher read density on chromosome 17 compared to other accessory chromosomes. D) Illumina sequencing of an excised chromosomal band at 0.9 Mb identified by PFGE in the offspring A2.2. Illumina sequencing reads were mapped to coding sequences of all chromosomes of the reference genome IPO323. E) Schematic illustration of the hypothesized non-allelic homologous recombination between inverted repeats that generated chromosome 17 in offspring A2.2 and A66.2. The resulting isodicentric chromosome can initiate a breakage-fusion-bridge cycle while the acentric chromosome will be lost during successive rounds of cell division.

In order to elucidate the origin of the novel chromosome found in two offspring of isolates 1A5 and 1E4, we performed whole genome resequencing of these progeny. The sequencing reads were mapped to all coding sequences of the reference genome, identically to the procedure used for the resequencing of the Swiss population. The parental isolates 1A5 and 1E4 both carried an almost complete set of accessory chromosomes except that 1E4 lacked chromosome 17 (Figure 3). Progeny A2.2 and A66.2 both showed a complete set of accessory chromosomes. However, in contrast to parental isolate 1A5, we did not find any mapping reads for coding sequences spanning the terminal portion of chromosome 17 (ranging from 481–558 kb on the reference chromosome 17; Figure 10A). This missing chromosome segment would result in a reduced length of approximately 100 kb compared to the length of chromosome 17 in the reference strain (full length 584 kb; [46]).

To test for potential duplication events, we used read depth on chromosome 17 as a proxy for duplicated sequences. The parental isolate 1A5 showed a homogeneous distribution of read depth along the chromosome. The parental isolate is suggested to be missing a large chromosomal segment between 1–85 kb compared to the reference genome (Figure 10B). The two progeny A2.2 and A66.2 also lacked the region between 1–85 kb compared to the reference genome (Figure 10B). The central region of chromosome 17 was divided into two sharply distinct regions based on read depth. A region of high read depth between 85–350 kb and a region of low read depth between 350–481 kb (Figure 10B).

We tested whether the increased read depth on chromosome 17 was distinct from the read depth on other chromosomes of the progeny. We calculated the average read density on coding sequences across all 13 core chromosomes as a reference baseline. The average read densities of the parental isolate 1A5 and the two progeny A2.2 and A66.2 were respectively 11.96, 30.93 and 36.34 reads per base pair of coding sequence. We compared these average values to read densities on accessory chromosomes (Figure 10C). In order to mitigate biases introduced by large missing segments on the various accessory chromosomes, we calculated the average read density using only mapped positions for each isolate. Accessory chromosomes of the parental isolate 1A5 showed read densities ranging from 69.4–94.4% of the average read density on core chromosomes, with chromosome 17 showing the lowest read density. Both progeny showed on average slightly higher read densities ranging from 69.1–122% and 76.1–133% for A2.2 and A66.2, respectively. Among all accessory chromosomes, chromosome 17 showed the largest increase (1.77–1.92 fold) in relative read density compared to the parental isolate 1A5 in both progeny (Figure 10C). We hypothesized that this nearly two-fold increase in read density reflected a large duplication event occurring on chromosome 17.

To determine the genomic content of the novel chromosomal band found in the two offspring, we excised the new chromosomal band found at 0.9 Mb from the PFGE gel of progeny A2.2. After purification and whole-genome amplification, we performed Illumina sequencing on the resulting amplified DNA. The sequencing reads were mapped to all coding sequences of the reference genome. The average read density per chromosome was highly variable, with most chromosomes showing an average read density of 0.78–17.4 reads per base pair (Figure 10D). By far the highest read density was found for coding sequences on chromosome 17 with 175 reads per base pair.

We designed two genomic probes specific to chromosome 17 and hybridized the probes to chromosomal bands separated by PFGE. The probes showed that parental isolate 1A5 was carrying a chromosome 17 of the expected length as compared to the reference isolate IPO323 (Figure 9B). We found no hybridization signal on any chromosomal band for parental isolate 1E4. Both progeny A2.2 and A66.2 showed a specific hybridization signal for chromosome 17 on the novel chromosomal band at 0.9 Mb (Figures 9B and 9C; probe 4 see Table 2). A second chromosome-specific probe for chromosome 17 gave identical results (data not shown). Taken together, this strongly suggests that the novel chromosome band is either entirely or almost entirely constituted by sequences belonging to chromosome 17.

## Discussion

We showed that the accessory chromosomes of *Z. tritici* underwent significant structural rearrangements including fusions and large insertions. The chromosomal complement is highly plastic with substantial variation both in the number of accessory chromosomes carried by each isolate and variation in gene content among homologous chromosomes. We located the exact breakpoints of multiple insertions in chromosome 14 that led to a drastic chromosome length polymorphism within a population. Meiosis played a significant role in shaping accessory chromosome complements. Segregation of some accessory chromosomes was distorted, with nondisjunction during meiosis leading to frequent losses of accessory chromosomes. We found evidence for the fusion of sister chromatids of chromosome 17 in two offspring from the same cross. These progeny carried a nearly doubled chromosome 17 generated through a chromosomal fusion in a subtelomeric region that was likely initiating a breakage-fusion-bridge cycle.

### Effects of population structure on global chromosomal diversity

The global survey of chromosomal segments revealed highly diverse accessory chromosome complements. We found that isolates not only differed in the number of accessory chromosomes as expected, but that homologous chromosomes showed markedly different gene contents due to numerous insertions and deletions. Several accessory chromosomes such as chromosome 16 were found near fixation in some populations, such as Australia and Israel. In contrast we found that chromosome 18 was almost entirely missing from the sampled Australian population. The near fixation or losses of accessory chromosomes in some populations may be due to stochastic processes such as founder events during the establishment of the pathogen in previously unaffected geographical regions. Populations also differed strongly in the diversity of chromosomal haplotypes detected by the PCR assays. The Swiss population had a much higher number of unique haplotypes for chromosomes 14, 16 and 18 than the Australian population. Founder effects were hypothesized to explain the low genetic diversity found for neutral markers in Australian *Z. tritici* populations that were introduced along with wheat not later than ∼200 years ago [50]. In agreement with this earlier finding, accessory chromosomal segments of the Australian population showed the strongest deviation from global frequencies. However, large variations in accessory chromosome complements were also found in other populations. Hence, the diversity in chromosomal complements reflects a previously uncharacterized form of genetic differentiation in this pathogen. Frequency differences in accessory chromosomes among populations may also result from selection operating on chromosomes carrying genes that confer a selective advantage or disadvantage in particular environments. For example, gene products such as effectors that contribute to host virulence in a gene-for-gene interaction may be strongly disfavored in some wheat fields due to the presence of matching resistance genes [54]. If virulence factors such as effectors are located on accessory chromosomes, this may enable rapid adaptation in an arms race to overcome detection by the host immune system. The rapid loss of non-essential virulence factors located on accessory chromosomes may provide a significant selective advantage to a fungal pathogen [32].

### Accessory chromosomes showed extensive variation in gene content

The resequencing of Swiss isolates revealed extensive variation in gene content among homologous accessory chromosomes. In comparison to the chromosome sequence of the reference strain, accessory chromosomes of the resequenced isolates carried deletions ranging from a few genes to large sections affecting several dozens of genes. Surprisingly, missing segments were rarely contiguous as would be expected from single deletion and insertion events generating a chromosomal length polymorphism. Accessory chromosome 16 showed numerous short deletions spanning only a few coding sequences in the Swiss population compared to the reference chromosome. Our resequencing analysis (Figure 3) suggests that several chromosomes may be missing chromosomal ends including telomeres. However, our resequencing data was not informative on the integrity of telomeric repeats and we could not be certain that telomeres were missing in these isolates. In the reference genome, one telomere sequence on chromosome 21 could not be sequenced and may be missing [46]. Intact telomeres play a crucial role in chromosomal stability by ensuring homologous chromosomal pairing and disjunction during meiosis [17]. Defective telomeres are thought to initiate the development of breakage-fusion-bridge cycles leading to major chromosomal anomalies [17], [18]. If some accessory chromosomes are indeed defective for telomeres, this may play a major role in generating the observed chromosome polymorphisms.

### Large insertion led to visible chromosomal length polymorphism

The most dramatic chromosomal length polymorphism segregating within a population was found for chromosome 14, with the shortest identified chromosome variant approximately half the length of the longest known chromosome variant. In a related pathogen found on barley (*Z. passerinii*) that is ancestral to the more closely related pathogens found on wild grasses [55] we identified a homologous chromosome. The ancestral form of chromosome 14 is largely identical to the shortest variant found in Iranian and Swiss populations. Several lines of evidence suggest that the large insertions leading to the chromosome 14 variant found in the reference strain occurred recently. First, the longest chromosome variant is found almost exclusively in the Swiss population, which is closest to the location where the Dutch reference isolate IPO323 was isolated. The A26b isolate from Iran was sampled close to the center of origin of *Z. tritici* and this isolate carried the shortest known chromosomal variant. Second, sequences immediately adjacent to the insertion breakpoint locations showed only a single nucleotide polymorphism compared to the reference chromosome. Third, sequences near the breakpoint location were highly similar even when compared with the phylogenetically distant *Z. passerinii*.

A major open question is the source of the inserted sequences. We did not find closely related sequences either at a different location in the reference genome or in any resequenced strain. The largest insertion in chromosome 14 contains several dozen genes and may have functional consequences for isolates carrying the large chromosome variant, because several of these genes were predicted to encode transcription factors or other functions. All three inserted sequences are flanked on at least one end by remnants of the same class of transposable elements. The presence of these elements near the flanking regions suggests that non-allelic homologous recombination with an unknown chromosome may have played a role in the insertion of these sequences into chromosome 14. Interestingly, the two shorter insertions in chromosome 14 showed a markedly lower GC-content than surrounding regions and these inserts were virtually devoid of genes. These isochores may be regions of reduced recombination, as the inserted regions may lack homologous sequences necessary for meiotic crossing-over. The largest inserted sequence also contains a very large palindromic sequence similar in extent to palindromes on the human Y chromosome [56], [57]. The palindrome is flanked by the remnants of two copies of a transposable element, similar to the inserted sequences. In yeast, palindromes were shown to mediate gene amplification and intra-chromosomal recombination and may lead to genomic instability [58], [59]. Goodwin et al. [46] hypothesized that accessory chromosomes originated through an ancient horizontal transfer from an unknown donor species. Our analyses show that the accessory chromosome 14 was maintained through multiple speciation events and hence may be a remnant of an ancient core chromosome. The large insertions observed in extant *Z. tritici* populations suggest that chromosome 14 is undergoing a degeneration process. The insertions do not seem to have a severe effect on the fitness of the organism, as many different length variants of chromosome 14 were found segregating within the Swiss population. The tolerance to large sequence rearrangements may be a hallmark of the degeneration process affecting accessory chromosomes.

### Segregation distortion and chromosomal loss during meiosis

Meiosis is thought to play a major role in genomic instability in fungi [28], [48], [60]. Non-allelic homologous recombination among dispersed repeats was hypothesized to be the main source of chromosomal length polymorphism in fungi [28], [60]. In *Z. tritici*, aberrations during meiosis were suggested to lead to the loss of accessory chromosomes and hence contribute to chromosomal number polymorphism among isolates [48]. Our analyses of progeny from three different crosses showed that chromosomal loss affected nearly all accessory chromosomes. We detected low levels of chromosomal losses for all accessory chromosomes except chromosome 15. The loss of a chromosome may be due to nondisjunction of sister chromatids during meiosis. This defect during meiosis would create progeny carrying a duplicated (i.e. disomic) chromosome. Frequent loss of accessory chromosomes during meiosis poses an apparent paradox in *Z. tritici*. Populations would be expected to gradually lose all accessory chromosomes over generations in the absence of mechanisms to maintain the accessory chromosomes. Interestingly, our data on inheritance of accessory chromosomes revealed a mechanism that may maintain accessory chromosome complements in populations. Analyses of segregation frequencies revealed that chromosomes 15 and 21, if present in only one of the two parental strains, were inherited significantly more frequently than expected under random segregation of the chromosomes. Distorted segregation was restricted to one cross and no distortion was detected in the second cross differing in the presence of chromosomes 15 and 21. Segregation distortion was found to be a key characteristic of numerous animal B chromosomes [61]. We hypothesize that segregation distortion is one of the mechanisms that maintains accessory chromosomes in *Z. tritici* populations.

### Chromosomal fusion can initiate breakage-fusion-bridge cycles

The most striking example of chromosomal plasticity was the fusion of sister chromatids to generate a much longer chromosome 17 in two progeny of Cross 2 (Figure 10). This meiotic abnormality occurred in a cross between a strain carrying a chromosome 17 similar to the reference isolate and a strain lacking the entire chromosome. One mechanism for creating the new chromosome is non-allelic homologous recombination between inverted repeats on sister chromatids ([62], Figure 10E). The recombination event could create an isodicentric chromosome 17 carrying duplicated and non-duplicated regions, consistent with the striking difference in read depth observed across chromosome 17 (Figure 10B). If the fused chromosome contains two centromeres, it is expected to form a bridge at anaphase and undergo BFB cycles [20], [21], [26]. The rejoining of broken ends during new rounds of cell division will create new chromosomal arrangements including deletions and duplications. The lack of a homologous chromosome 17 during meiosis may have contributed to the initiation of a BFB cycle. The fate of the novel chromosome over subsequent generations is currently under investigation. The meiotic pairing of the large duplicated chromosome 17 with the parental chromosome variant is likely to generate further highly unstable chromosomal variants. *Z. tritici* possesses a genomic defense mechanism known as RIP [46] that is common to a large number of ascomycete fungi [63]. RIP rapidly degenerates highly similar genomic regions through the introduction of point mutations. We predict that the novel chromosome 17 variant generated by duplicating a large fraction of the original chromosome would be subjected to rapid degeneration as a result of RIP. We propose that BFB cycles coupled with RIP played a major role in creating the degenerated accessory chromosomes of *Z. tritici*.

### Rapid chromosomal structure evolution drives diversity in accessory chromosomes

Our study revealed extensive yet viable chromosomal rearrangements generated by meiosis. Genomic instability and insertion of exogenous sequences led to highly diversified sets of homologous chromosomes affecting hundreds of genes. The large number of insertions and deletions found among accessory chromosomes suggests that these chromosomes underwent an extensive degeneration process. The chromosomal degeneration process may well have been initiated in an ancestor of *Z. tritici*. The shorter gene length and lower gene density on accessory chromosomes compared to core chromosomes suggests that degeneration processes affected accessory chromosomes over long evolutionary time scales. We identified large insertions and the initiation of breakage-fusion-bridge cycles as two major contributors to chromosomal abnormalities. Surprisingly, isolates of *Z. tritici* appear to be highly tolerant of these abnormalities, which may contribute to the maintenance of extensive karyotypic diversity in populations. The extensive degeneration, distorted segregation and frequent loss of accessory chromosomes highlight a central question surrounding fungal accessory chromosomes: How and when do these chromosomes originate? We showed that chromosome 14 is ancient, as its origin predates several speciation events prior to the emergence of *Z. tritici* in the Fertile Crescent [55]. We postulate that the accessory chromosomes found in extant *Z. tritici* populations likely originated from the core chromosomes through a degeneration process. The initiation of chromosome degeneration is particularly likely in isolates that carry disomic chromosomes due to nondisjunction. Disomy would provide redundancy in gene content and, hence, relax selection pressure to maintain chromosomal integrity. Chromosomal degeneration may then proceed rapidly through BFB cycles, nondisjunction and RIP of duplicated regions. The emergence of a highly diverse and rapidly evolving set of accessory chromosomes in *Z. tritici* illustrates how an accessory genome can be created to serve as a cradle for adaptive evolution in this and other fungal pathogens.

## Materials and Methods

### Populations and isolates included in the study

We assessed the diversity in chromosomal structure in a global collection of *Z. tritici*. We included field populations from Israel (n = 23), Oregon, USA (n = 19), Switzerland (n = 26) and Australia (n = 30) (Table S2). These populations were previously assayed for neutral genetic diversity and variation in quantitative traits [53]. These isolates showed substantial variation for several quantitative characters, including virulence, fungicide resistance and thermal adaptation, among and within populations [51]–[53], [64].

### Establishment of sexual crosses

Sexual crosses were performed between three pairs of isolates from the Swiss population (see Table S2) using the established protocol for *Z. tritici* [65]. The crosses were between isolates ST99CH9B8B and ST99CH9G4C (Cross 1), ST99CH1A5 and ST99CH1E4 (Cross 2) and ST99CH1A5 and ST99CH3D7 (Cross 3).

### Chromosomal segment PCR assay

In order to survey presence-absence polymorphism among accessory chromosomes, we designed PCR assays to amplify approximately 500 bp of coding sequences at regular intervals of approximately 100 kb along the chromosomes of reference strain IPO323. For detailed information on the targeted genes and chromosomal locations see Table S1. Primers for PCR amplification were designed on conserved sections of the targeted coding sequence. Sequence conservation was assessed using the reference assembly of nine resequenced Swiss isolates and two resequenced Iranian isolates (for details see below). We used Primer 3.0 for primer design [66]. In order to control for successful PCR, we included a primer pair of a microsatellite locus in each PCR mix [67]. Successful PCRs produced a band at approximately 250 bp that was clearly distinguishable from the PCR product associated with each chromosomal segment. PCR reactions were performed in 20 µl volumes containing approximately 5–10 ng genomic DNA, 0.5 µM of each primer, 0.25 mM dNTP, 0.6 U Taq polymerase (DreamTaq, Thermo Fisher, Inc.) and the corresponding PCR buffer. PCR products were visualized on agarose gels.

### Plotting and analyses of chromosomal segment PCR assays

We used the R graphics package ggplot2 to plot the raw datasets and analyses [68], [69]. Measures of genetic differentiation among populations (F_ST_) were calculated with the function var.comp in the R package hierfstat [70]. The presence-absence data generated by the PCR assays were considered as two possible alleles at haploid loci. We tested for segregation distortion of chromosomal segments among progeny by testing for deviations from the expected 1∶1 segregation ratio of presence-absence polymorphism among progeny with a χ^2^ contingency table. We accounted for non-independence of chromosomal segments and multiple testing with a conservative Bonferroni correction. We calculated the repeat content on accessory chromosomes by identifying direct repeats with a repeat motif between 2–50 bp [71]. For each repeat, we calculated the total length of the repeat and subtracted the number of mismatches in the repeat motif, as a proxy for the extent and purity of the repeat element.

### Genome resequencing of *Z. tritici* isolates

We used the previously published genome assemblies of two Iranian isolates of *Z. tritici* ST01IRA26b and ST01IRA48b. In addition, we included five genomes of *Z. pseudotritici* (STIR04_3.11.1, STIR04_2.2.1, STIR04_4.3.1, STIR04_5.3, STIR04_5.9.1) four genomes of *Z. ardabiliae* (STIR04_3.3.2, STIR04_3.13.1, STIR04_1.1.1, STIR04_1.1.2) and one genome of the outgroup species *Z. passerinii* (P63) [72], [73]. All genome assemblies are available under the NCBI BioProject [PRJNA63131] on GenBank. We resequenced nine *Z. tritici* isolates from Switzerland (full isolate names: ST99CH1A5, ST99CH1E4, ST99CH3B8, ST99CH3C4, ST99CH3D1, ST99CH3D7, ST99CH3F5, ST99CH9B8B and ST99CH9G4C) and two progeny from Cross 2 (A2.2 and A66.2). We performed Illumina paired-end sequencing on 500–700 bp insert libraries to generate between 1–2 Gb of quality-trimmed sequence data per isolate (theoretical coverage of 25–50×). The read length was either 82 bp or 90 bp. Illumina sequence data are available from the NCBI Short Read Archive (see Table 1 for accessions).

### Genome de novo assembly

We used SOAPdenovo v. 1.5 [74] to generate de novo assemblies, including scaffolding and gap closing. De novo assemblies yielded a scaffold N50 ranging from 79,920–121,161 bp depending on the resequenced isolate. Total assembly space (scaffolds and singletons) ranged from 35.57–38.33 Mb (see Table 1). All genome assemblies are available on GenBank under BioProject [PRJNA178194] (see Table 1). The comparison with the total finished genome size for the reference isolate IPO323 (39.7 Mb) shows that the genomic assemblies account for a very large proportion of the genome of the sequenced isolates. The assembly statistics were similar to the assemblies reported earlier for the same species [72].

### Illumina read assembly on the reference genome

We mapped the Illumina reads of each resequenced isolate and offspring to the finished genome of IPO323 [46]. We used Bowtie 2.1.0 [75] to perform the mapping, allowing only reads that were mapped as paired-ends. We assessed the read coverage on the reference genome by filtering all reads based on their mapping quality (minimum mapping quality of 20) with GATK version 2.3-9-ge5ebf34 [76]. Coverage of coding sequences was extracted using the BEDtools utilities [77]. We scored the absence of coding sequences conservatively, requiring that less than 10 bp of a coding sequence should be covered and that the average read density on the coding sequence would be below 2×.

### Alignment of genomic scaffolds

Structural changes among chromosomes of different isolates were analyzed using Nucmer [78]. We used the –mum option requiring unique anchor matches that are unique in both the query and the reference genome. Genome assemblies were compared in pairwise comparisons between the finished reference genome of IPO323 and the draft assemblies of the different isolates of *Z. tritici*, *Z. pseudotritici*, *Z. ardabiliae* and *Z. passerinii*. In order to visualize synteny among different variants of chromosome 14, we extracted all scaffolds matching the reference chromosome 14. We discarded scaffolds that were shorter than 10 kb and that showed a match identity with the reference chromosome of less than 80%. Scaffold alignments were plotted with the R package ggplot2 [69]. Repetitive and palindromic sequences of the reference chromosome 14 of IPO323 were visualized by performing a self-alignment with LASTZ (http://www.bx.psu.edu/~rsharris/lastz).

### Characterization of chromosome 14

The finished chromosome 14 sequences were analyzed for short and medium length tandem repeats with the software Tandem Repeat Finder v. 4.04 [79]. We set the matching weight to 2, the mismatching and indel penalty to 10 and the match and indel probability to 80 and 10, respectively. The minimum alignment score was required to be 10 and the maximum period size of repeats was set to 50 bp. The occurrence of repeats was visualized along a 5 kb sliding window (with increments of 1 kb). The gene density on each chromosome was reported as the occurrence of start codons according to the latest annotation [46]. GC content of each chromosome was reported in 5 kb sliding windows with increments of 1 kb. We identified transposable element remnants on chromosome 14 by querying the annotated repeat libraries provided by Repbase Update [80].

### Preparation of fungal material for molecular karyotyping

High molecular weight chromosomal DNA (Ch-DNA) was prepared by *in situ* digestion of cell walls of agarose-embedded conidia. We used a slightly modified non-protoplasting method according to McCluskey et al. [81]. The following Z. *tritici* isolates were used: ST01IRA26b, ST99CH9B8B (parental isolate of Cross 1), ST99CH9G4C (parental isolate of Cross 1), ST99CH1A5 (parental isolate of Cross 2 and 3), ST99CH1E4 (parental isolate of Cross 2), ST99CH3B8, ST99CH3C4, ST99CH3D1, ST99CH3D7, ST99CH3F5 and IPO323. In addition, we included the isolate P63 of *Z. passerinii* [55]. To screen progeny of sexual crosses, we randomly selected 24 and 34 confirmed progeny from Cross 1 and Cross 2, respectively.

All isolates were transferred from stocks maintained in glycerol at −80°C to Yeast Malt Agar (YMA) plates and were grown for 3 to 4 days in the dark at 18°C. After incubation, conidia were washed off the plates with sterile water and 600–800 µl of suspended conidia were transferred to 2 to 3 fresh YMA plates. The plates were incubated for 2 to 3 days as described above. Conidia were harvested using sterile distilled water and filtered through sterile Miracloth (Calbiochem, La Jolla CA, USA) into 50 ml screw-cap Falcon tubes. The tubes were filled with distilled water up to 50 ml total volume. The suspension was centrifuged at 3750 rpm at room temperature for 15 min with a clinical centrifuge (Allegra X-12R, Beckman Coulter, Brea CA, USA). The resulting pellets were resuspended in 1–3 ml TE buffer (10 mM Tris-HCL, pH 7.5; 1 mM EDTA, pH 8.0) and gently vortexed. The spore concentration of the solution was determined using a Thoma haematocytometer cell counter. An aliquot of 1.5 ml spore suspension with a concentration between 8×10^7^ to 2×10^8^ spores/ml was transferred to a fresh 50 ml screw-cap tube and incubated at 55°C in a water bath for several minutes. To each tube, 1.5 ml pre-warmed (55°C) low-melting-point agarose prepared in TE Buffer was added (2% w/v; molecular biology grade, Biofinex, Switzerland). The solution was thoroughly mixed by gentle pipetting. An aliquot of 500 µl was solidified on ice for approximately 10 min in a precooled plug casting mold (BioRad Laboratories, Switzerland). A total of five agarose plugs per isolate were incubated in 15 ml screw-top tubes containing 5 ml of a lysing solution containing 0.25 M EDTA, pH 8.0, 1.5 mg/mL protease XIV (Sigma, St. Louis MO, USA), 1.0% sodium dodecyl sulfate (Fluka, Switzerland). The incubation was performed for 28 h at 55°C. During the incubation the lysing solution was changed once after 18 h and gently mixed every 2–3 h. Chromosomal plugs were washed three times for 15–20 min in 5–6 ml of a 0.1 M EDTA (pH 9.0) solution and then stored in the same solution at 4°C until they were used.

### Pulsed-field gel electrophoresis

Pulsed-field gel electrophoresis (PFGE) was carried out using a BioRad CHEF II apparatus (BioRad Laboratories, Hercules CA, USA). Chromosomal plugs were inserted into the wells of a 1.2% and 1.0% (wt/vol) agarose gel (Invitrogen, Switzerland) to separate small chromosome (<1 Mb) and medium-sized chromosomes (1.0 Mb–3.0 Mb), respectively. Small chromosomes (i.e. accessory chromosomes) were separated at 13°C in 0.5× Tris-borate-EDTA Buffer (Sambrook & Russell 2001) at 200 V with a 60–120 s pulse time gradient for 24–26 h. Medium-sized chromosomes were separated at 100 V with a 250–900 s pulse time gradient for 48–50 h using the same buffer and running temperature as above. Gels were stained in ethidium bromide (0.5 µg/ml) for 30 min immediately after the run. Destaining was performed in water for 5–10 min. Photographs were taken under ultraviolet light with a Molecular Imager (Gel Doc XR+, BioRad, Switzerland). As size standards, we used chromosome preparations of *Saccharomyces cerevisiae* (BioRad, Switzerland) and *Hansenula wingei* (BioRad, Switzerland).

### Southern transfer and hybridization of pulsed-field gels

Southern blotting and hybridization were performed according to standard protocols [82]. In summary, hydrolysis was performed in 0.25 M HCl for 30 min and DNA was blotted onto Amersham HybondTM-N+ membranes (GE Healthcare, Switzerland) overnight under alkaline conditions [82]. DNA was fixed onto the membranes at 80°C for 2 h. Membranes were prehybridized overnight with 25 ml of a buffer containing 20% (w/v) SDS, 10% BSA, 0.5 M EDTA (pH 8.0), 1 M sodium phosphate (pH 7.2) and 0.5 ml of sonicated fish sperm solution (Roche Diagnostics, Switzerland). Probes were labeled with ^32^P by nick translation (New England Biolabs, Inc.) following the manufacturer's instructions. Hybridization was performed overnight at 65°C. Blots were subjected to stringent wash conditions with a first wash in 1× SSC and 0.1% SDS and a second wash with 0.2× SSC and 0.1% SDS. Both washes were performed at 60°C. Membranes were exposed to X-ray film (Kodak BioMax MS) for 2 to 3 days at −80°C. All hybridization probes used to identify specific chromosomes are listed in Table 2.

### Excision of chromosomal band and amplification

Chromosomal DNA was separated with CHEF gel electrophoresis as previously described for the separation of small chromosomes except that a 1.0% agarose gel was used. The novel 0.9 Mb chromosomal band from isolate A2.2 was excised and DNA was recovered using the Wizard SV Gel and PCR Clean-up System kit (Promega, Switzerland) with the following modifications to the manufacturer's recommendations: during the incubation at 65°C the gel slice was vortexed two times for 5 minutes, sonication was for 3 min and followed by a final incubation for 1 min. The resulting purified DNA was amplified using a whole genome amplification kit (REPLI-g Mini Kit, Qiagen, Germany). Amplified DNA was subjected to whole genome sequencing with an Illumina HiSeq 2000 as described above.

## Supporting Information

Figure S1 Global survey of diversity in accessory chromosomes of *Zymoseptoria tritici*. The presence or absence of chromosomal segments were assayed by PCR in a global collection of four field populations located in Australia, Israel, United States and Switzerland (total n = 98). Horizontal rows indicate different isolates included in the study. Green and red rectangles indicate the presence and absence, respectively, of a chromosomal segment assayed by PCR. Locations of individual PCR assays are indicated in Figure 1 and Supplementary Table S1. Core chromosomes 10 and 13 were included for comparison with the accessory chromosomes 14–21.
(EPS)

Table S1 Chromosomal position, gene identifier and primer sequences for the PCR assay on core and accessory chromosomes.
(DOCX)

Table S2 Global collection of *Zymoseptoria tritici* isolates included in the study.
(DOCX)
