# Supplementary material for: Breakage-fusion-bridge Cycles and Large Insertions Contribute to the Rapid Evolution of Accessory Chromosomes in a Fungal Pathogen
Source: PLoS Genet. 2013 Jun 13;9(6):e1003567. doi: 10.1371/journal.pgen.1003567 (PMC3681731; doi:10.1371/journal.pgen.1003567)
Supplement: Table S1 — Chromosomal position, gene identifier and primer sequences for the PCR assay on core and accessory chromosomes. (DOCX) [file pgen.1003567.s002.docx]

**Table S1:** Chromosomal location, gene identifier and primer sequences for the conserved PCR assay on core chromosomes 10 and 13, and accessory chromosome 14-21. The position corresponds to the start of the exon in the reference genome IPO323 [1]. The gene identifier refers to the corresponding protein ID.

| Chromosome | Position | Gene, exon | Forward primer (5'-3') | Reverse primer (5'-3') |
| --- | --- | --- | --- | --- |
| 10 | 99,793 | 67705, exon 2 | ACTACCTCGATGTCGTCTACTCC | AACATGAAGTTCTGGTATGGGAT |
| 10 | 305,532 | 54658, exon 1 | TACAAAGCAGAGCAATCTGTCAT | CAGAAGCCATTTTCTTGACAAC |
| 10 | 502,373 | 107851, exon 1 | CAATGGAAACAGCTTATCACAAA | TGTCATCAATCTCTTCCAGATAGC |
| 10 | 725,541 | 36274, exon 2 | ATTCCTGGCGACCAACTATGT | GTTTGGTTGCCTTCGAGTTT |
| 10 | 923,899 | 67987, exon 1 | CTGAAAGAGGATATGCTTGGTATG | CTTCTTGAGATGGTCTCATTCAGT |
| 10 | 1,176,574 | 107958, exon 1 | CCACGAGACGTAGATACATACTTTG | GTTTCTACACTGTGGAGGCTTTCT |
| 10 | 1,317,437 | 107981, exon 2 | GAGCAATACAGCTAAATGGACAAC | ATAGTATGCCAGGCGTTTGAAG |
| 10 | 1,536,480 | 55182, exon 1 | GCTACGTCTTTCCCATAATACCAC | CAGCTTGTAGCTTGGATATGAGAT |
| 13 | 54,148 | 88369, exon 3 | AATGCTTCTTATTGTGGACGAAG | ACCTGATCTGAAGTAATGGTGATG |
| 13 | 168,740 | 111591, exon 1 | TACCAATCAACACATCAGCAAATA | CCTGAGTTCAGTGACGAATAGTGT |
| 13 | 311,772 | 51250, exon 5 | TCTTCTTGAGTACAAAAAGGCACT | GTCCTTCCAATCTCTTCCATCTT |
| 13 | 499,550 | 77796, exon 1 | CGCAAGAGAACTACAACTCCTACT | GCTTTCCTGTAAAGACTGAATGTC |
| 13 | 620,955 | 97402, exon 2 | CGATCAGCAATATCTCCATAGTCT | GTCCTGGACACACTGTAAGAAATG |
| 13 | 811,448 | 51025, exon 2 | GCGACATGAAAAACTACATCAAAG | GATCTCAGGAAGGAGATTCGTC |
| 13 | 998,966 | 78014, exon 1 | TCTGGACTACAAACTGAACTTTCG | TCAAACTCAAAGTCCGTCTCAG |
| 14 | 80,172 | 97536, exon 2 | ATCATTAAGTTCCTCTTCCTCCTC | GCTTCCTCTTCCACTTGCTC |
| 14 | 127,225 | 111731, exon 1 | CAAGGCAGGCCTTATTCTTTC | ATAGAACGAGAAGATGTTGCTGAG |
| 14 | 177,891 | 97554, exon 1 | CTCCTCTCCGTTGTCCTACTTG | GACTTGGCGGAGGTAGAAGAC |
| 14 | 211,772 | 97562, exon 1 | GTATCGCTAGTGCGTTTATCGAC | GAGATGGTGTGGATATCTTGGAT |
| 14 | 260,182 | 97566, exon 1 | AAATTGAGCAGATGGTTACATTCA | TTGGACAAGATATCAAAGGACACT |
| 14 | 298,634 | 97571, exon 2 | TACCAAGGAGACTGTCCTATTCAG | ATTGAGAAGGATCTCACACTTCTG |
| 14 | 327,085 | 51592, exon 6 | ACAGTATCGAATGTTGGTACGTTT | AAGAATGATAAGACCTTTGTGCAG |
| 14 | 369,872 | 97585, exon 3 | GAGACGATATCGGCATTCTAAAC | CTGTTCTGTAAGGGGAAGTGTTC |
| 14 | 485,840 | 78038, exon 4 | ATCATCTTAGCTTATGTGCTCTGG | CTATCCCTCTACGTCTTTCTCCC |
| 14 | 573,748 | 51580, exon 2 | AATACAGCGTTAAGTGTTCCTTTG | GTGAGTCTTGTAACAGTCGTGTGA |
| 14 | 605,988 | 51634, exon 1 | CAGAACTGGACCGAGTATTTACAG | CCAGGTCTGATACACAAAAAGTTC |
| 14 | 647,947 | 111740, exon 5 | AGGCTTTCGAAGACATTGAATC | CCTGCAGCAGCCAACTCT |
| 14 | 679,456 | 97610, exon 2 | GCTCTGAGTACCTGCATACATACC | TGACAGAGCTATATTCGTCTGGAT |
| 14 | 735,539 | 97616, exon 1 | ATTTGGTATGAAGAACCAGCCT | GATTTGACGTAGTGGAAGGAGTTA |
| 15 | 33,563 | 97617, exon 5 | CTGGACGATCTTCACTACCACTAT | GAAGAATCTTGACGGACGAAAG |
| 15 | 114,771 | 97624, exon 2 | AGTACGTCAGCAAGAACGACTTC | CGATCGACGTATCCATGTAGTT |
| 15 | 277,211 | 97646, exon 1 | GGAATTATCTCTCCTGGACTTGAT | TGGGATTCTTGAGTTGTTGTCTAC |
| 15 | 358,554 | 97661, exon 5 | AAATACTAAGCGGCAAACCAC | GAGTTTGGGAGTTGTTGAAGTTG |
| 15 | 431,764 | 97674, exon 1 | ACTTCCACAAGTCTCAGATGGAT | GAAGAGTGATAGAAGACCACAAGG |
| 15 | 477,423 | 97683, exon 1 | GACATATGGCACTACGAAGCTC | CTTCGATGTCAGACTCTTCAGTG |
| 15 | 556655 | 111755, exon 3 | CACATCCGACATTAACAACAAGTA | CGCGTTTGCAAAGTAGACAC |
| 16 | 62,513 | 51708, exon 1 | GTGTTCCTGTACCTCAAGTCTACG | TACTGAGCCACTTTCTCAAAATCT |
| 16 | 107,640 | 97707, exon 2 | CCATCACACCTCCGTACTATTG | CTCAGACTTTTCTGAAGGAAGAGG |
| 16 | 153,430 | 97717, exon 5 | CCTGGAGAATTCAAGAATAGGTTT | GCTGATACTGAACCAGAGACTCCT |
| 16 | 239,791 | 97729, exon 1 | AGGCTCCATGTGCAACTAGAA | GAAATTGGCACGGATGATCT |
| 16 | 309,196 | 97739, exon 5 | AGCTACGCTCAGTTTCGTACAG | GGTTTCGAAATGCGGATCTAT |
| 16 | 358,108 | 97748, exon 3 | CATAGATCTCCGTCAACGATGT | CATATCGACTGCGTCTTGACT |
| 16 | 403,443 | 97759, exon 2 | GAGCATGAGACTAGTGGAGATTGT | GGCTGCTCGATGAGATACTGT |
| 16 | 486,116 | 97768, exon 1 | GAAAATCAGCACCTGACAAGG | ATACGAAGTTGCAGATGAAAGGT |
| 17 | 16,108 | 97779, exon 1 | GTTACTGGGCAACATCAAGACTAC | GACGTGTGCTTATTTTGTACCTTC |
| 17 | 141,772 | 97793, exon 1 | ACTACTGAAAGGGCAGAGTACGTT | GATGCGACGTCTCGTGTCT |
| 17 | 224,581 | 97809, exon 1 | GCCATCTACCACAAAGCTCTC | GTTTGGAGATGGAGATGGAGAT |
| 17 | 363,049 | 97830, exon 2 | AAGTACAAGAGCGAGAGATTCCTT | CAATGAGTGCACGAAGCTG |
| 17 | 474,992 | 97838, exon 2 | CCAATCCCAAGAAAACCG | GACCTTTTGTGAGCTTCTCAAGTA |
| 17 | 557,313 | 111767, exon 2 | ACTTTATCAACTGCGGTGTCTT | GTACTCCTCCGTCTTCTGCTG |
| 18 | 59,051 | 97857, exon 3 | CGATCAAGAGTGCAAGGTATTCT | AGTCACCACACTTCAGAACGAC |
| 18 | 107,086 | 97862, exon 2 | ACACTATGATTGGTGACTCTTGCT | AATGGACCTTGAGATAAATCTTCG |
| 18 | 216,737 | 97880, exon 1 | GAGGATCTTCTCAGTGTCGATG | GTTGTTTTCCTTCTCATGGTCTC |
| 18 | 329,347 | 111776, exon 1 | GGATCCTCTCCACCACTACTATG | ACCCACCCACTACTTGAGAGAC |
| 18 | 401,544 | 97896, exon 1 | AGAAGATTTCCTCTCCGATTCTC | GTGGAAACGTTGGATGTGATT |
| 18 | 521,830 | 97910, exon 4 | GACTTCATCCTGTCGTACACCTT | GTGCTTGTACTTGTTGTTGAGAGT |
| 19 | 46,980 | 97921, exon 1 | AAAGATATGAGCGAAGACGACAG | AGGCACACACTTCCGTGTC |
| 19 | 144,896 | 97935, exon 1 | GATGGAACGTGTACTCACGACT | GTCGTCTTCTGTCTTGAACGTATT |
| 19 | 216,690 | 97948, exon 1 | TCTATAACCTCCACGAGGATGAA | TCTTTCTTCCTCCCACATACAG |
| 19 | 296,040 | 97971, exon 2 | GAAGACTCCCTTGCTCGTCT | CATCTCCTCCCTCTTGGACTT |
| 19 | 397,380 | 97980, exon 1 | AAGATAACGAGGAAAGCAACG | CGAAGGCCTCCGTAAGAGT |
| 19 | 522,250 | 97994, exon 1 | GCTATGATACAGAGGGCACATAAC | ACTGGGTCCGACATTTTCAT |
| 20 | 56,844 | 98006, exon 1 | GCCTATAGACACCTCCTACGTCTA | CATCCGAGTCTGATTCCTTGT |
| 20 | 136,087 | 98024, exon 1 | AACCTATCTCGAGAATCACAACG | GTCTTGTAGACCGTCGTCTCCT |
| 20 | 190,818 | 98032, exon 3 | GAAAAGAAGGCCACCTTGAG | ACTGATTTCCAAATCGGAGAAG |
| 20 | 279,286 | 111800, exon 1 | CTTTATTGACGGACATTCACTTCT | AATCTGCTGATTCTGCTTCTTCT |
| 20 | 365,027 | 98061, exon 1 | GGAGTCCAAGCTCTTACCATATCT | AAGATGCAAAGTTTCGCGTT |
| 20 | 445,118 | 98072, exon 1 | GAGTACTCTGACGAGCATGGC | GAAGACCTCGTCCATCTTCAG |
| 21 | 54,775 | 98076, exon 1 | CTGAGCGTGGAAGTAACCAA | ATGAACACAGCACACTTCTTGTC |
| 21 | 195,328 | 111806, exon 8 | AAATTGGAGGAAAAGAACAATGAG | GATCACCGTACATACCAACGTC |
| 21 | 306,203 | 111815, exon 6 | GTCGAATTGGTTGAAATCAGTG | ATGTTGTCTATCTCCTGGTTGATG |
| 21 | 358,584 | 98124, exon 2 | ATACCAAGAACGACAGCGATATT | ATCAACCAAGTAGATGGTGGTG |

1. Goodwin SB, M'barek SB, Wittenberg AHJ, Crane CF, Hane JK, et al. (2011) Finished genome of the fungal wheat pathogen *Mycosphaerella graminicola* reveals dispensome structure, chromosome plasticity, and stealth pathogenesis. PLoS Genet 7: e1002070. doi:10.1371/journal.pgen.1002070.
