# Supplementary material for: Breakage-fusion-bridge Cycles and Large Insertions Contribute to the Rapid Evolution of Accessory Chromosomes in a Fungal Pathogen
Source: PLoS Genet. 2013 Jun 13;9(6):e1003567. doi: 10.1371/journal.pgen.1003567 (PMC3681731; doi:10.1371/journal.pgen.1003567)
Supplement: Table S2 — Global collection of Zymoseptoria tritici isolates included in the study. (DOCX) [file pgen.1003567.s003.docx]

**Table S2:** Global collection of *Zymoseptoria tritici* isolates included in the study.

| Isolate | Abbreviated isolate name | Population | Year of collection / publication | Strain used for crosses |
| --- | --- | --- | --- | --- |
| STIR01A26b | A26b | Iran | [1] |  |
| STIR01A48b | A48b | Iran | [1] |  |
| ST99CH3A1 |  | Switzerland | [1] |  |
| ST99CH3A2 |  | Switzerland | [1] |  |
| ST99CH3A4 |  | Switzerland | [1] |  |
| ST99CH3A5 |  | Switzerland | [1] |  |
| ST99CH3A6 |  | Switzerland | [1] |  |
| ST99CH3A9 |  | Switzerland | [1] |  |
| ST99CH3A10 |  | Switzerland | [1] |  |
| ST99CH3B1 |  | Switzerland | [1] |  |
| ST99CH3B2 |  | Switzerland | [1] |  |
| ST99CH3B4 |  | Switzerland | [1] |  |
| ST99CH3B8 | 3B8 | Switzerland | [1] |  |
| ST99CH3C1 |  | Switzerland | [1] |  |
| ST99CH3C4 |  | Switzerland | [1] |  |
| ST99CH3C7 | 3C7 | Switzerland | [1] |  |
| ST99CH3D1 | 3D1 | Switzerland | [1] |  |
| ST99CH3D3 |  | Switzerland | [1] |  |
| ST99CH3D5 |  | Switzerland | [1] |  |
| ST99CH3D7 | 3D7 | Switzerland | [1] | Cross 3 |
| ST99CH3D8 |  | Switzerland | [1] |  |
| ST99CH3F1 |  | Switzerland | [1] |  |
| ST99CH3F2 |  | Switzerland | [1] |  |
| ST99CH3F5 | 3F5 | Switzerland | [1] |  |
| ST99CH1A5 | 1A5 | Switzerland | 1999 | Cross 2 and Cross 3 |
| ST99CH1E4 | 1E4 | Switzerland | 1999 | Cross 2 |
| ST99CH9G4C | 9G4C | Switzerland | 1999 | Cross 1 |
| ST99CH9B8B | 9B8B | Switzerland | 1999 | Cross 1 |
| STa12-3B.2 |  | USA | [1] |  |
| STa12-3B.3 |  | USA | [1] |  |
| STa12-3B.6 |  | USA | [1] |  |
| STa12-3B.7 |  | USA | [1] |  |
| STa12-3B.8 |  | USA | [1] |  |
| STa12-3B.9 |  | USA | [1] |  |
| STa12-3B.10 |  | USA | [1] |  |
| STa12-3B.11 |  | USA | [1] |  |
| STa15-3B.19 |  | USA | [1] |  |
| STa15-4A.2 |  | USA | [1] |  |
| STa15-4A.3 |  | USA | [1] |  |
| STa15-4A.4 |  | USA | [1] |  |
| STa15-4A.7 |  | USA | [1] |  |
| STa15-4A.10 |  | USA | [1] |  |
| STa15-4A.11 |  | USA | [1] |  |
| STa15-4A.13 |  | USA | [1] |  |
| STa15-4A.15 |  | USA | [1] |  |
| STa15-4A.17 |  | USA | [1] |  |
| STa15-4A.19 |  | USA | [1] |  |
| ISYAr1b |  | Israel | [1] |  |
| ISYAr1c |  | Israel | [1] |  |
| ISYAr1j |  | Israel | [1] |  |
| ISYAr2b |  | Israel | [1] |  |
| ISYAr2f |  | Israel | [1] |  |
| ISYAr4e |  | Israel | [1] |  |
| ISYAr4f |  | Israel | [1] |  |
| ISYAr4g |  | Israel | [1] |  |
| ISYAr5g |  | Israel | [1] |  |
| ISYAR8d |  | Israel | [1] |  |
| ISYAr12d |  | Israel | [1] |  |
| ISYAr12e |  | Israel | [1] |  |
| ISYAr12f |  | Israel | [1] |  |
| ISYAr15c |  | Israel | [1] |  |
| ISYAr16a |  | Israel | [1] |  |
| ISYAr16h |  | Israel | [1] |  |
| ISYAr17b |  | Israel | [1] |  |
| ISYAr17e |  | Israel | [1] |  |
| ISYAr17i |  | Israel | [1] |  |
| ISYAr18b |  | Israel | [1] |  |
| ISYAr19e |  | Israel | [1] |  |
| ISYAr21a |  | Israel | [1] |  |
| ISYAr22f |  | Israel | [1] |  |
| ST01Aus1A6 |  | Australia | [1] |  |
| ST01Aus1A5 |  | Australia | [1] |  |
| ST01Aus1A9 |  | Australia | [1] |  |
| ST01Aus1B1 |  | Australia | [1] |  |
| ST01Aus1B7 |  | Australia | [1] |  |
| ST01Aus1B8 |  | Australia | [1] |  |
| ST01Aus1B2 |  | Australia | [1] |  |
| ST01Aus1C3 |  | Australia | [1] |  |
| ST01Aus1C1 |  | Australia | [1] |  |
| ST01Aus1C6 |  | Australia | [1] |  |
| ST01Aus1C8 |  | Australia | [1] |  |
| ST01Aus1C2 |  | Australia | [1] |  |
| ST01Aus1C7 |  | Australia | [1] |  |
| ST01Aus1D5 |  | Australia | [1] |  |
| ST01Aus1D9 |  | Australia | [1] |  |
| ST01Aus1D4 |  | Australia | [1] |  |
| ST01Aus1E1 |  | Australia | [1] |  |
| ST01Aus1E4 |  | Australia | [1] |  |
| ST01Aus1E5 |  | Australia | [1] |  |
| ST01Aus1F8 |  | Australia | [1] |  |
| ST01Aus1F3 |  | Australia | [1] |  |
| ST01Aus1F2 |  | Australia | [1] |  |
| ST01Aus1G2 |  | Australia | [1] |  |
| ST01Aus1G5 |  | Australia | [1] |  |
| ST01Aus1H1 |  | Australia | [1] |  |
| ST01Aus1D8 |  | Australia | [1] |  |
| ST01Aus1H2 |  | Australia | [1] |  |
| ST01Aus1H6 |  | Australia | [1] |  |
| ST01Aus1H8 |  | Australia | [1] |  |
| ST01Aus1H9 |  | Australia | [1] |  |

1. Zhan J, Linde CC, Jürgens T, Merz U, Steinebrunner F, et al. (2005) Variation for neutral markers is correlated with variation for quantitative traits in the plant pathogenic fungus *Mycosphaerella graminicola.* Mol Ecol 14: 2683–2693. doi:10.1111/j.1365-294X.2005.02638.x.
